# A Comprehensive Review of the Characteristics Associated with Lightweight Cement

**DOI:** 10.1021/acsomega.5c09092

**Published:** 2026-03-30

**Authors:** Seyyed-Mohammad-Mehdi Hosseini, Mohammad Ranjbar, Hasan Maroof, Reza Zabihi, Mehdi Ostadhassan, Mahin Schaffie

**Affiliations:** † Department of Petroleum Engineering, 48511 Shahid Bahonar University of Kerman, University Blvd., 22 Bahman Street, 76169-13439 Kerman, Iran; ‡ Mineral Industries Research Center, 48511 Shahid Bahonar University of Kerman, University Blvd., 22 Bahman Street, 76169-13439 Kerman, Iran; § Department of Petroleum Engineering, University of Tehran, Enghelab Ave., 14174-66191 Tehran, Iran; ∥ Institute of Geosciences, Marine and Land Geomechanics and Geotechnics, Christian-Albrechts-Universitat, Ludewig-Meyn-Strasse 10, 24118 Kiel, Germany

## Abstract

Effective cementing in deep and weak formations is crucial for maintaining good integrity, particularly when the fracture pressure margin is minimal. This work explores both experimental and practical applications of various lightweight cements and presents key findings. The achieved slurry densities range from 1000 to 2200 kg/m^3^, with compressive strengths reaching up to 72 MPa. The inclusion of zeolite at levels of 5% to 25% by weight of cement (BWOC) consistently reduces the clinker content by at least 30%. Additionally, zeolite increases water demand, enhances the gel structure, and facilitates the rapid development of strength. Metakaolin, utilized at concentrations ranging from 10% to 20%, improves mechanical properties and durability; however, higher dosages may prolong thickening time, requiring optimization of cobinders. Vermiculite retains strength at high temperatures (up to 1650 °F), reduces thermal conductivity, and enhances plugging efficiency in fractured rock. Gilsonite provides waterproofing, stability, and long-term durability with minimal water requirements. Using perlite at approximately 4% BWOC reduces plastic viscosity by about 30%, increases yield point by around 330%, and can enhance compressive strength by up to 88%. Furthermore, waste expanded perlite can boost strength by roughly 50% while decreasing CO_2_ emissions. Ground granulated blast-furnace slag (GGBS) at 30% BWOC optimizes the density-strength balance and reduces the permeability by approximately 50%, with field trials reporting a 33% reduction in CO_2_ emissions. Hollow glass microspheres and cenospheres achieve densities of about 1200–1600 kg/m^3^, with a moderate strength reduction (10–15%) beyond 30% inclusion. Silica fume (5–15%) enhances long-term strength and resistance in CO_2_-rich or marine environments. Foamed cement systems allow for extreme lightweighting (approximately 1000–1300 kg/m^3^), decreasing gas migration rates by about 60%. Cross-comparisons identify optimal blends such as SF + GGBS or MK + zeolite for creating stable, lightweight matrices. The adoption of these additives supports sustainability by reducing cement use and CO_2_ emissions by 25–40%, aligning lightweight cementing practices with both performance and environmental objectives.

## Graphical abstract

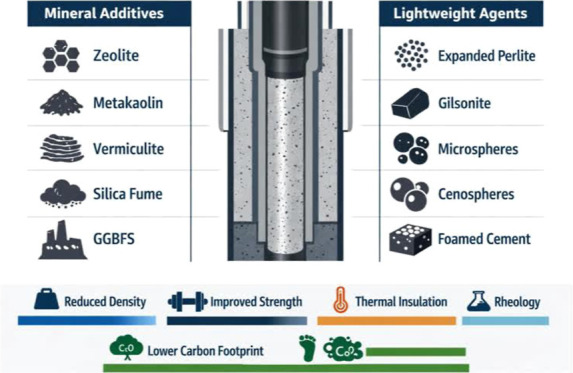

## 1 Introduction

Drilling deep wells and navigating weak formations are challenges that every drilling engineer faces. Hence, cementing operations represent a critical stage in drilling and completing oil and gas wells.^1–3^ Operational failures can lead to significant financial losses due to the tight gap between fracture pressure and pore pressure in the depleted zone, along with weak formations, increasing the risk to well production.^4–6^ Narrow tolerances in weak formations mean that variations in drilling parameters significantly impact borehole conditions, often leading to fluid loss during drilling and cementing in low-breakdown-pressure or deep reservoirs.^7^ Therefore, effective cementing in weak formations is vital, requiring careful consideration of the cement density throughout the process. This attention ensures the integrity and safety of wells, protecting investments and the environment.^8–10^ The American Petroleum Institute (API) categorizes cement wells into eight groups, labeled from A to H, along with three levels of sulfate resistance: high, ordinary, and moderate.^11–13^ Additionally, various additives are incorporated into slurry formulations to improve the performance across diverse conditions. Accelerators are additives that reduce thickening time and promote early strength gain, such as calcium chloride.^14,15^ Retarders, such as lignosulfonate, are another valuable additive used to prolong the cement thickening time, ensuring sufficient time for slurry placement. Extender additives reduce cement slurry density (e.g., microspheres), while weighting materials increase it (e.g., hematite).^16^ Fluid loss additives, like starch, reduce water loss to formations, and dispersants lower slurry viscosity for better flow, while defoamers, including nonionic surfactants and silicon derivatives, minimize air in concrete.^17–20^ Placing cement in the annulus space can be challenging when dealing with unstable formations.^21^ However, the final slurry composition is up to many factors, like downhole temperatures, fracture gradient, and fluid pressure.^22^ Using lightweight cement has substantially revolutionized the durability of oil and gas wells and diminished the costs associated with well maintenance.^23^ More importantly, using appropriate additives can greatly decrease greenhouse gas emissions released during the production of cement, which is considered an extremely harmful process, leading to positive long-term effects on various environmental aspects.^24^ Drilling engineers often express concern about the lower compressive strength of this type of cement compared to conventional systems. This highlights the importance of carefully evaluating cement selection to ensure the durability and effectiveness of drilling operations.^25–27^ This paper presents findings from experimental and field studies on the effectiveness of lightweight cements, especially in the context of well cement. It reviews efforts to reduce environmental pollution associated with cement production and usage through experimental and simulation methods. The discussion opens with the critical issue of lost circulation, which lightweight cement intends to address. Lost circulation happens when drilling fluid or cement slurry seeps into permeable formations during drilling and cementing operations.^28,29^ As shown in Figure 1, formations that are more susceptible to lost circulation include highly permeable zones, areas with caves and faults, as well as natural and induced fractures.^30^

**Figure 1:**
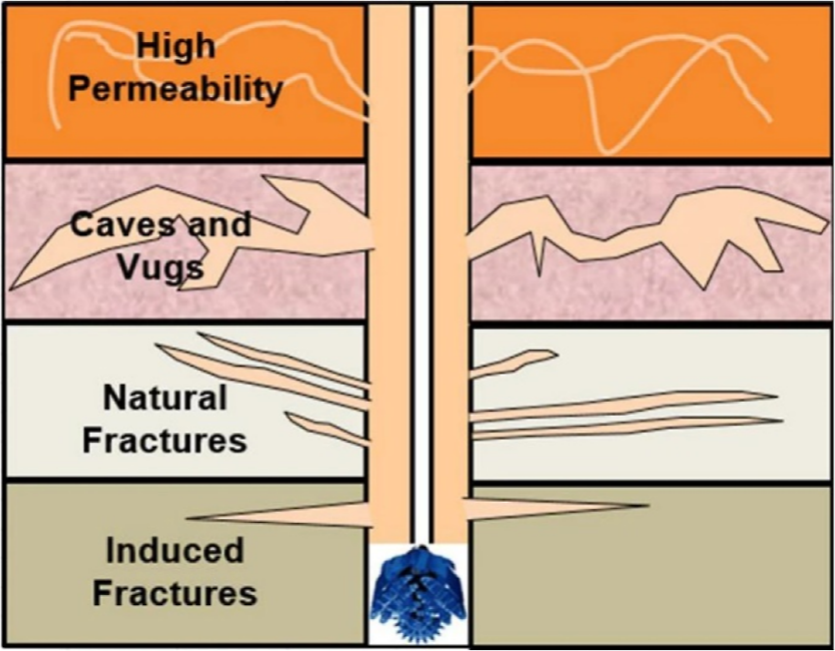
Formation prone to lost circulation. Reprinted from ref 31 under the Creative Commons Attribution 4.0 International (CC BY 4.0) license.

To mitigate the risk of lost circulation, a comprehensive strategy is essential, considering all relevant factors. This can be approached through two main strategies: corrective measures and preventive practices.^32^ Put simply, from a preventive perspective, managing return losses through proactive planning is indispensable to avoiding their occurrence. For instance, applying excessive overbalanced pressure can fracture the formation and result in losses.^33^ Therefore, to inhibit compressive failure of the rock, wellbore pressure should be maintained below the fracture pressure and above the optimal formation pressure.^34,35^ Furthermore, corrective action involves addressing lost circulation by using lost circulation materials (LCM) to treat this issue during drilling operations. In other words, these substances are added to the fluids to plug the loss zones.^36^ Lightweight cement is key to addressing issues in oil well operations, offering benefits like eliminating multistage tools, reducing nonproductive time and costs, and enhancing perforation characteristics compared to traditional neat cement.^37,38^ Incorporating natural substances to partially replace cement powder in slurry compositions presents a significant opportunity to reduce costs and simultaneously tackle environmental concerns by lowering CO_2_ emissions.^39^ This innovative method not only improves the sustainability of construction practices but also plays a role in fostering a greener future.^40^ From an operational standpoint, water-extended slurries offer easier on-site preparation and lower material cost but suffer from limited stability and narrower temperature tolerance, often requiring precise control of free water and mixing energy. In contrast, low-SG-extender systems (e.g., containing perlite, vermiculite, or HGMs) provide superior density control and gas-migration resistance, though they demand more complex blending, higher material handling care, and agitator modifications to prevent solid segregation.^41^ In the following, the results obtained from using standard lightweight cement systems with various additives are elaborated on. Some gained insight is mentioned in Table 1. Moreover, Figure 2, which is inspired by previous studies and provides an overview of the conceptual framework, enhances our understanding of the various aspects of lightweight cement, highlighting its advantages and disadvantages.

**Table 1:** Summary of Outcomes from Studies, Part 1

section	key points
drilling deep wells and navigating weak formations	√ this poses a significant challenge for a drilling engineer
	√ cementing operational failures can lead to major financial losses due to risks in the depleted zone
	√ ensuring proper cementing in weak formations, especially with proper density, is essential for well integrity and environmental safety
the most prevalent issue encountered during drilling operations is lost circulation	√ fluid loss occurs when drilling fluids penetrate porous geological formations, leading to expensive interruptions and safety issues
	√ areas with high permeability, caverns, fractures, and both natural and artificially created fissures are more prone to experiencing lost circulation
to reduce lost circulation, it is crucial to develop a detailed plan addressing all key operational factors	√ lightweight cement is crucial for oil well operations, providing benefits such as reducing nonproductive time and costs, enhancing perforation, and eliminating the need for multistage tools
	√ by replacing cement powder with natural materials in slurry mixtures, costs can be reduced, and CO_2_ emissions lowered, promoting sustainability in construction and a greener future

**Figure 2:**
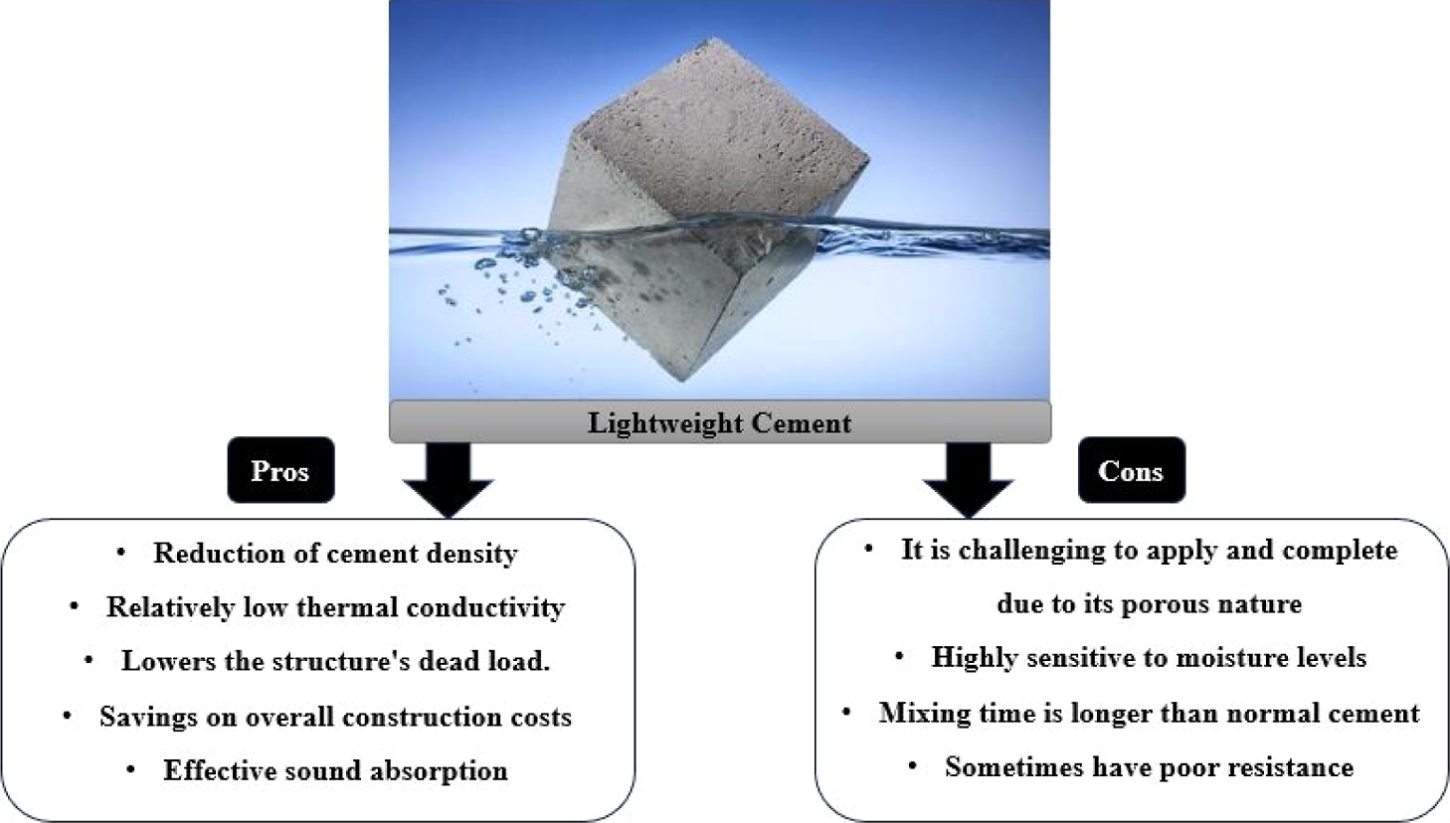
Overview of the pros and cons of lightweight cement. Conceptual framework adapted from ref 136 under the Creative Commons Attribution 4.0 International (CC BY 4.0) license.

## 2 Literature Search Strategy

To ensure a comprehensive review, a systematic literature search was conducted across major engineering databases, namely Scopus, Web of Science (WoS), and the specialized repository OnePetro, to ensure comprehensive coverage of relevant publications, covering the period from 1995 up to 2025. The primary search strings included combinations of terms such as (“Lightweight Cement” OR “Low Density Slurry”) AND (“Lost Circulation” OR “Wellbore Stability”). Inclusion criteria mandated that articles present experimental or simulation data for cementing weak formations. Exclusion criteria removed non-English papers, purely theoretical works lacking quantitative results, and articles focused exclusively on general cement chemistry unrelated to wellbore applications. The final selection process involved a two-stage screening (title/abstract review followed by a full-Text review).

### 2.1 Lightweight Cement Using Water

Various methods exist to reduce the cement density, with the most common being the addition of water. This process results in a type of lightweight cement known as water-extended cement.^42^ Increasing environmental concerns about the damage caused by raw material extraction and carbon dioxide emissions during cement production have inspired a collective effort to adopt supplementary materials and reduce cement consumption for a more sustainable future.^43^ Blends of Portland cement and pozzolan are frequently used in cement production to address environmental issues and economic benefits, as Larki et al.^23^ achieved valuable results in a study focused on creating a lightweight cement slurry with a density of 1680 kg/m^3^ (14 ppg), using a combination of natural pozzolan and Class G cement. The results showed that the optimal substitution of cement powder with pozzolan was 30%, as determined by 24 h compressive strength and water volume tests. A higher content of pozzolan impacted pumpability. To replicate bottom-hole conditions, the temperature was increased to 150 °F, and additives were used to maintain the slurry properties. The ideal concentrations for the fluid loss control agent, dispersant, and retarder were 0.5%, 0.08%, and 0.05% by weight of cement, respectively. After 30 days, the compressive strength reached 24.32 MPa.^23^ Further, many industries have made significant advancements in innovative cementitious materials, enhancing cement’s compressive strength and its ability to set and harden underwater. These improvements expand its applications and can lead to superior construction results and greater durability.^44,45^ Besides, bentonite material increases the viscosity of slurries; therefore, it is recommended to use it with a viscosity-reducing agent to achieve optimal performance.^23,46^ In addition, bentonite increases the ratio of water-to-cement (WCR) and can be mixed into cement using either dry blending or wet blending methods.^47^ In dry blending, bentonite and cement are mixed before water is added, which limits the cement dispersion and increases the bentonite demand. In wet mixing, bentonite is combined with water from the start, effectively separating particles and reducing the overall need for bentonite.^48^ For slurry requirements under 500 barrels, wet mixing is preferred. Excessive bentonite in cement can weaken the strength and prolong the thickening time. However, bentonite is beneficial in cement slurry production, helping to address environmental challenges and reduce pollution from cement manufacturing.^49^ Memon et al.^50^ studied the use of Pakistani bentonite as a partial cement replacement in concrete, with replacement levels of 5% to 20% by weight of cement (BWOC). Results indicated that higher bentonite content reduced workability due to its high surface area, but a 10–15% replacement improved compressive strength and durability through pozzolanic reactions and better particle packing. Beyond this range, the strength decreased. Additionally, bentonite reduced water absorption and permeability, enhancing resistance to sulfate attack. Furthermore, Farhan Mushtaq et al.^51^ explored the effects of bentonite as a partial cement replacement in concrete subjected to high temperatures. Concrete mixed with 5%, 10%, and 15% bentonite was tested at temperatures up to 800 °C, assessing compressive strength, density, water absorption, and microstructure. Results indicated that up to 10% bentonite enhanced the thermal stability and maintained compressive strength due to denser microstructures. However, higher levels led to a strength reduction from hydration phase decomposition. Bentonite mixes exhibited lower weight loss and reduced cracking compared to those of the control.

### 2.2 Lightweight Additives with Low Specific Gravity

Lightweight systems that use water extensions need a significant quantity of water, despite possessing weak mechanical properties. One alternative method for creating lightweight slurries is to utilize materials that have a specific gravity lower than that of traditional cement.^52^ Studies show that these additives reduce cement density while maintaining adequate compressive strength, which will be examined further.

#### 2.2.1 Zeolite

Zeolite is a microporous aluminosilicate with a low specific gravity (approximately 2400 kg/m^3^ (20 ppg)) used as a partial cement replacement or lightweight filler in the production of lightweight cement (LWC and lightweight oil well cement).^53^ Zeolite has the potential to significantly reduce the density of cement, bringing it down from approximately 2300 kg/m^3^ (19 ppg) to around 1600 kg/m^3^ (13 ppg), resulting in a weight reduction of about 30%. However, to achieve an optimal balance between reducing weight and maintaining structural strength, the ideal industrial usage generally falls within a range of 10–15% (BWOC).^54^ Understanding the framework structure is essential for understanding zeolite chemistry. Zeolites can be classified into two categories—natural and synthetic—based on their structural characteristics and the type of framework they possess.^55^ Moreover, a key element in classifying zeolite types is the framework type, which represents the connections among tetrahedrally coordinated atoms in the most symmetrical way possible.^56^ The framework type of zeolites significantly influences their molecular interactions. For instance, MOR and ZSM-5 zeolites are more likely to attract CO_2_ compared to USY and BEA types.^57^ In simple terms, each zeolite has a three-letter code for its framework type, composed of TO4 tetrahedra, mainly [AlO_4_] and [SiO_4_]. Shared oxygen atoms create connections between aluminum and silicon, while charge repulsion prevents Al–O–Al bonds. This structure forms cages linked by specific pore openings.^58^ The negative charge in the lattice is balanced by the positive charge of cations in the material’s pores. Zeolites are typically synthesized in hydrothermal conditions using autoclaves, starting from reactive gels in alkaline media at temperatures of 68 to 176 °F.^59,60^ Increasing cement production will undoubtedly lead to higher energy consumption and, consequently, serious environmental issues, particularly in terms of carbon dioxide emissions.^61^ Using natural pozzolans, such as zeolites, as a cement substitute are a valuable option for reducing cement consumption. Studies have evaluated zeolite’s performance in this role.^62,63^ Clinoptilolite is the primary natural zeolite, recognized for its unique structure. It contains water molecules and exchangeable cations, which exhibit pozzolanic properties similar to those of silica fume. In simple terms, these cations are considered superior to other materials found in synthetic products.^64^ Bilim investigated the characteristics and behavior of cement mortar that includes clinoptilolite, which is among the most prevalent zeolite minerals occurring in nature.^65^ Natural clinoptilolite was dried and ground to a fine powder (below 75 μm) and used as a partial replacement for Portland cement in a mortar at 5%, 10%, 15%, 20%, and 25% BWOC. Compressive strength was tested on days 7, 28, and 56, alongside assessments of water permeability, capillary absorption, and water absorption to evaluate pozzolanic effects. Incorporating clinoptilolite at 10–15% BWOC resulted in only 8–13% lower compressive strength than the control, while water permeability and capillary absorption improved by 15–20%. However, substitutions of 20% or more led to significant strength loss (around 18%) and a reduced flow. A medium dosage is recommended for lightweight, durable cement applications. The study’s strengths include its dosing design and simultaneous property testing, but it lacks microscopic analysis and long-term durability data, limiting its industrial applicability, such as in oil and gas well cementing. Valipour and his colleagues^66^ studied the environmental impact of green concrete with natural zeolite, focusing on the global warming index (GWI) under marine conditions. They replaced 15–30% of Portland cement with zeolite and evaluated the concrete’s durability and resistance to chlorides, also conducting a life cycle assessment (LCA). The findings showed that this substitution maintained or improved durability while significantly reducing CO_2_-equivalent emissions and global warming potential due to lower cement usage and zeolite’s pozzolanic activity. The study concluded that natural zeolite is an effective partial cement replacement, enhancing the sustainability of marine concrete structures. This study introduces a new approach to assessing the environmental impact of concrete that contains natural zeolite. However, it emphasizes the GWI too heavily and is limited to specific marine conditions. As a result, the findings may not be applicable to other environments. Hassan et al.^67^ investigated the production of eco-friendly cements using synthetic zeolite catalysts as pozzolanic materials to partially replace Portland cement. The synthetic zeolite, prepared from recycled aluminosilicate sources, enhanced the pozzolanic reactivity, leading to higher long-term compressive strength and a 20–30% reduction in greenhouse gas emissions compared to ordinary cement. While the study effectively demonstrated the chemical activity and sustainability potential of synthetic zeolite, its replacement range was limited, and no detailed energy-cost trade-off or long-term durability validation in aggressive environments (e.g., marine or sulfate exposure) was provided. Nonetheless, the work represents a notable advancement toward low-carbon cement systems integrating catalytic, high-reactivity zeolitic components. Then, Broni-Bediako et al.^68^ conducted an experimental evaluation of fresh nanozeolite as an additive to Class G oil-well cement following the API RP 10B-2 test protocols. They investigated dosages of 1%, 2%, and 3% (BWOC) at a water-to-cement ratio of 0.44. The incorporation of nanozeolite increased slurry viscosity by approximately 8% to 15% and reduced thickening time by 12% to 20%, leading to a faster set time. Besides, permeability decreased from (9.089 × 10^–17^ m^2^) 0.092 mD in the control sample to (6.022 × 10^–17^ m^2^) 0.061 mD at a 2% dosage, while the compressive strength improved from 28.5 MPa in the control sample (cured for 72 h at 140 °F) to 33.1 MPa at the 2% nanozeolite dosage, representing a 16% increase. Microstructural analysis revealed a denser calcium-silicate-hydrate (C–S–H) gel and fewer capillary voids, confirming that nanozeolite effectively enhances early strength, reduces fluid flow, and improves durability under high-temperature, high-pressure well conditions. The addition of fresh nanozeolite enhances the cement slurries’ carrying capacity (plastic viscosity to yield point ratio), as illustrated in Figure 3. Although the results of the study at 3% BWOC dosage prove the practical value and short-term improvement of OWC, to generalize to real industrial conditions and high-pressure and high-temperature (HPHT), the dose range and tests should be broadened and deepened, and long-term phase and durability analyses should be added.

**Figure 3:**
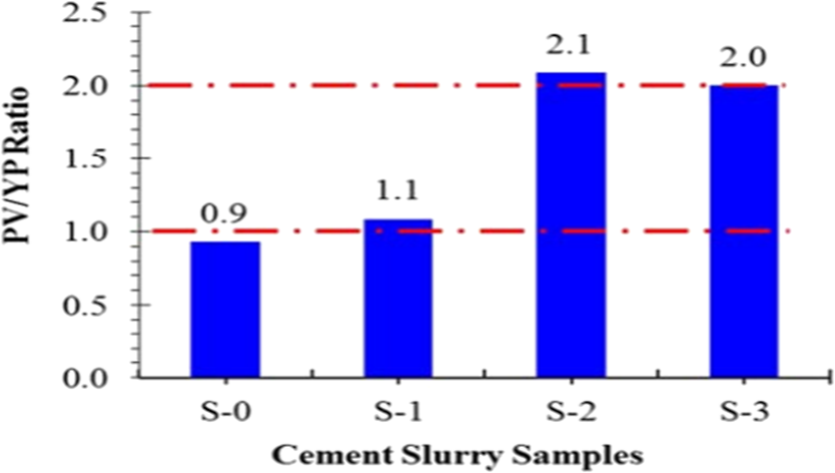
Ability of the slurry to carry various concentrations of fresh nanozeolite at 80 °F. Reprinted from ref 68 under the terms of the Creative Commons Attribution 4.0 International (CC BY 4.0) license.

Kriptavičius et al.^69^ investigated the combined effects of natural zeolite and ground glass as partial replacements for Portland cement on the durability of concrete. Their study found that a mixture containing 10% zeolite and 10% glass (a total of 20% replacement) exhibited optimal performance. Specifically, water absorption decreased by approximately 25%, chloride penetration depth was reduced by around 35%, and the compressive strength at 28 days increased from 47.2 MPa (control) to 49.8 MPa, representing a 5% increase. After 120 days of exposure to a Na_2_SO_4_ solution, the mass loss decreased by approximately 40%, demonstrating enhanced sulfate resistance. Microstructural analysis revealed a denser (C–S–H) gel enriched with alumina and a reduction in capillary pores. However, while the quantitative durability results were thorough, the discussion of the reaction mechanisms remained qualitative and did not include thermal or kinetic modeling to validate the long-term synergy between zeolite and glass across varying cement compositions.

As summarized in Table 2 studies indicate that zeolite, including natural clinoptilolite, synthetic forms, and nanozeolite, is a trending additive for oil well cement (OWC), enhancing mechanical performance and stability. All of them show a 5–15% decrease in slurry density and a 5–18% increase in water requirement due to the porous structure and high specific surface area of zeolite, which aids in well pressure control. API 10 B standard tests in recent studies (2019–2023) have shown an average thickening time of 3 to 3.5 h at BHCT (Bottom Hole Circulating Temperature) temperatures of 113–158 °F. The initial compressive strength was 25–40 MPa but increased to 50–60 MPa within 72 h, indicating a gradual pozzolanic reaction and the formation of more stable C–S–H gel phases. In terms of stability, most of the new compositions had free water of less than 1% and negligible sedimentation, and the permeability was reduced by up to 70%, effectively inhibiting gas migration. From an economic standpoint, the cost of using zeolites in these compounds depends on the type of zeolite. On average, prices range from approximately dollars 35 per ton for natural zeolite to around dollars 120 per ton for nano- or synthetic zeolites.

**Table 2:** Summary of Zeolite Studies

year	reference	density (g/cm^3^)	water demand change	thickening time (API 10B, h)	compressive strength (MPa) at 24/72 h (BHCT/BHST)	stability (free water/sedimentation)	HPHT range (°F/MPa)	estimated cost (dollar/t)
2010	Rahhal et al.^64^	2.00–2.15	↑ 5–8%	2.8–3.0	38/52 (at BHCT 185 °F/BHST 248 °F)	<1% FW; negligible sediment	230 °F/30 MPa	≈dollars 35–40
2011	Bilim^65^	1.95–2.20	↑ 10–12%	3.2–3.4	42/60 (at BHCT 185 °F/BHST 248 °F)	<0.8%; stable	248 °F/35 MPa	≈dollars 45
2014	Valipour et al.^66^	1.75–2.00	↑ 10–15%	3.0–3.2	45/65 (at BHCT 185 °F/BHST 248 °F)	<1%; consistent	266–275 °F/35 MPa	≈dollars 40–60
2019	Hassan et al.^67^	1.80–2.05	↑ 7–10%	2.9–3.1	48/70 (at BHCT 194 °F/BHST 266 °F)	<0.5%; excellent	275–283 °F/38 MPa	≈dollars 60–80 (synthetic)
2021	Broni-Bediako & Naatu^68^	1.70–1.85	↑ 12–15%	3.3 ± 0.2	50/72 (at BHCT 212 °F/BHST 275 °F)	<0.5%; steady	284–304 °F/40 MPa	≈dollars 90–120 (nano)
2023	Kriptavičius et al.^69^	1.90–2.05	↑ 8–10%	3.0–3.5	55/68 (at BHCT 203 °F/BHST 266 °F)	<0.5–0.8%; uniform	275–284 °F/40 MPa	≈dollars 55–70

#### 2.2.2 Metakaolin

Metakaolin is one of the most active synthetic pozzolanic materials in the cement and concrete industry, obtained by calcining kaolin at a temperature of 1202–1472 °F. Its key feature is the quick reaction with Ca­(OH)_2_, leading to the formation of secondary phases C–S–H and C–A–H that significantly enhance the physical and chemical properties of cement paste. Metakaolin, known for its high pozzolanic activity, helps reduce the porosity and permeability while minimizing thermal resistance loss. This, in turn, enhances the durability of the concrete. In the context of lightweight and oil well cements, metakaolin plays a crucial role in stabilizing rheology, controlling gas, and increasing strength under (HPHT) conditions.^70,71^ Metakaolin is not inherently lighter than Portland cement; however, due to its high reactivity and fine-grained structure, it helps to reduce the effective density of the slurry while increasing strength at lower densities (approximately 1500–1650 k/m^3^) (12.5–14 ppg). As a result, it is widely used in lightweight cement applications and in oil well construction.^72^ Brooks et al.^73^ examined the effects of chemical admixtures on the thickening time of high-strength concrete (compressive strength >60 MPa) using water-to-cement ratios of approximately 0.30–0.32. They found that polycarboxylate superplasticizers slightly delayed the initial setting time by 5–10 min, while lignosulfonates caused significant delays of up to 80 min. In contrast, calcium chloride (CaCl_2_) accelerated the thickening time, reducing it by 30–40%. The study linked admixture chemistry to hydration kinetics, providing a framework for managing setting behavior in dense cement systems. However, it did not explore strength development at later ages or interactions of multiple admixtures, limiting its applicability to complex field-scale concrete applications. Additionally, a recent study evaluated the feasibility of using a particular type of metakaolin.^74^ In fact, researchers evaluated Qusaiba kaolinitic shale from Saudi Arabia as a supplementary cementitious material (SCM) in lightweight Class G oil-well cement formulations. The shale was thermally activated at 1382 °F to enhance pozzolanic reactivity, and cement replacement levels of 5%, 10%, and 15% were tested under curing conditions of 302 °F and 45 MPa for 72 h. Results showed that at 10% replacement, the slurry density decreased from 1920 kg/m^3^ (16.0 ppg) to 1820 kg·m^–3^ (15.2 ppg), while the compressive strength after 72 h increased by about 18% (from 26.8 to 31.6 MPa), with the water permeability decreasing by 22% and the thickening time extending slightly by about 12 min. Scanning electron microscopy (SEM) analysis evidenced denser C–S–H and Al-substituted gel phases responsible for higher compactness and durability. Although the quantitative outcomes and thermal activation methodology are robust, the study lacks comparison with benchmark SCMs (e.g., GGBS) and omits long-term thermal/hydrothermal cycle testing, which limits the translational reliability of results for downhole high-temperature applications. Then, Lou and his colleagues once^75^ examined the metakaolin-based alkali-activated cement’s compressive strength over 28 days, finding that the compressive strength of geopolymer cement from metakaolin is influenced by the Si/Al ratio and activator concentration. It was also stated that when utilizing a low-reactivity metakaolin as a precursor, it is essential to incorporate more reactive cobinders—reactive metakaolin and Ground Granulated Blast Furnace Slag (GGBFS)—into the mixture. These cobinders help promote early strength by facilitating the formation of K-A-S-H and C-A-S-H gels, and they also enhance the dissolution of the low-reactive metakaolin, further contributing to strength development. Both cobinders exhibited similar trends in strength development; however, generally, higher strength values were noted when GGBFS was used as a cobinder compared to metakaolin. Furthermore, Ahmed et al.^76^ concentrated on the use of kaolinite as an additive and have shown its potential to enhance the performance of high-density cement by altering essential properties. The study investigated various concentrations of kaolinite and its effects on several properties, including rheology, thickening time, porosity, permeability, and compressive strength. The introduction of 1% BWOC kaolinite significantly reduced both settling and permeability, slightly decreased porosity, increased compressive strength, improved rheological properties, and extended the thickening time. Figure 4 displays the cement’s compressive strength with the kaolinite concentration. As it is known, a 1% concentration of kaolinite BWOC reduces particle settling by 74.4% and increases compressive strength by 13%. Furthermore, kaolinite enhanced rheological characteristics by reducing plastic viscosity by 8.4%, increasing the yield point by 19.4%, and boosting gel strength by 30%. Plus, kaolinite reduced plastic viscosity by 8.4% compared to the baseline cement while increasing yield point and gel strength by 19.4% and 30%, respectively. In cement systems with heavy-weight additions, including kaolinite, particle settling was reduced, leading to a density variation of 0.26% compared to 1% in the base sample. Besides, nuclear magnetic resonance (NMR) analysis confirmed the capability of kaolinite to reduce the settling of hematite particles, as demonstrated by consistent porosity distributions throughout the kaolinite-modified cement samples. Additionally, Figure 5 shows how permeability and porosity vary with changes in kaolinite concentration. As shown, higher concentrations of kaolinite resulted in decreases in both permeability and porosity, suggesting strong pore-filling abilities, and lowered permeability and porosity by 74% and 7%, respectively. Given the importance of environmental issues, Abiodun et al.^77^ examined the mechanical properties and durability of metakaolin in concrete production. They found that undissolved quartz in metakaolin enhances compressive strength and that its high silica (93.39%) and alumina contents contribute to strong pozzolanic and geopolymer properties. Metakaolin’s significant absorption capability necessitates the use of a superplasticizer, as increased water content can weaken the concrete. Mixtures with 15 wt % metakaolin showed the highest compressive strengths, attributed to increased CSH, and a reduced water-to-cement ratio further improved strength. Geopolymer concrete with metakaolin exhibited an 11.5% enhancement in compressive strength at 28 days compared with traditional concrete and had significantly higher water absorption capabilities. Additionally, using metakaolin lowers manufacturing temperatures and carbon emissions, potentially reducing CO_2_ emissions by 70% to 100%. It can decrease the cement density from approximately 2250–2600 kg/m^3^ (19–13 ppg), with a typical reduction of 10–15% in practice, resulting in a final density between 1550 and 1650 kg/m^3^ (13–14 ppg).^78^

**Figure 4:**
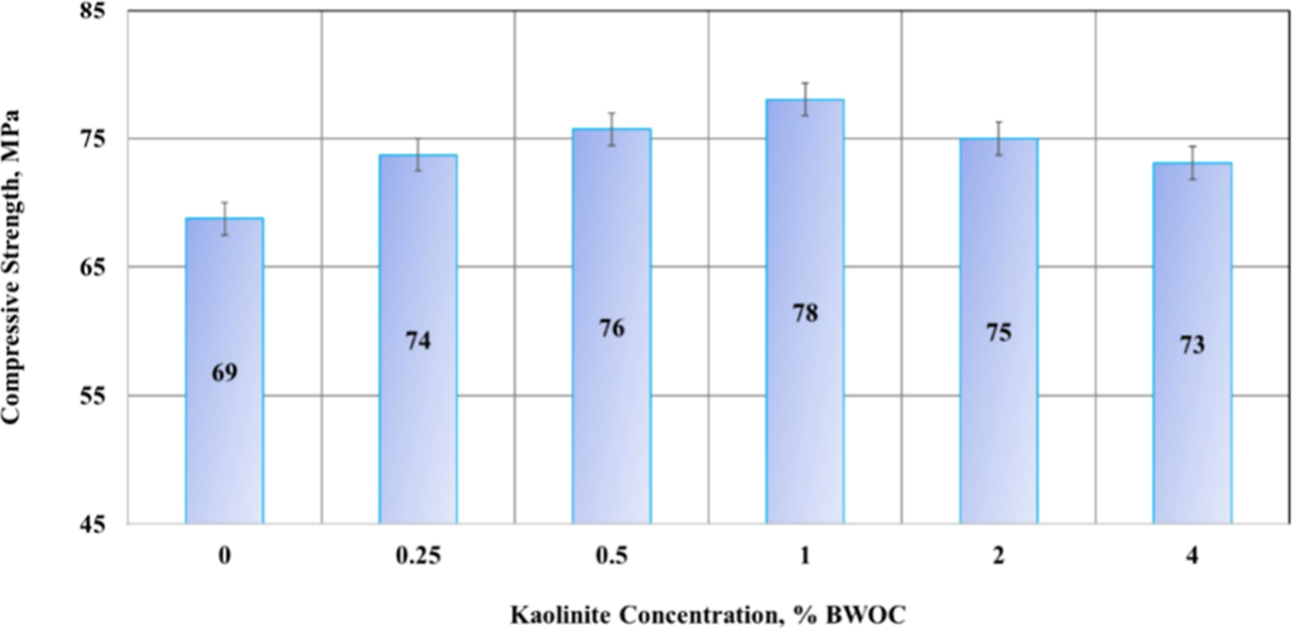
Outcomes of the compressive strength. Reprinted from ref 76 under the Creative Commons Attribution 4.0 International (CC BY 4.0) license.

**Figure 5:**
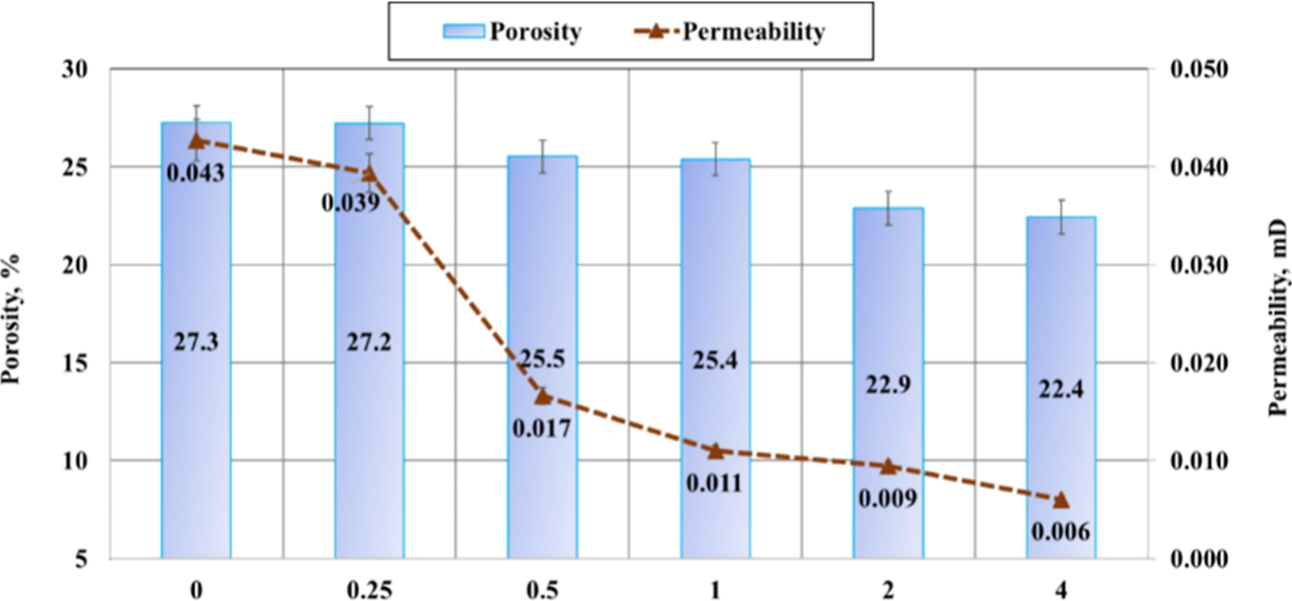
Outcomes of porosity and permeability. Reprinted from ref 76 under the Creative Commons Attribution 4.0 International (CC BY 4.0) license.

In general, metakaolin-based studies show (Table 3) that these additives improve slurry mechanical properties and stability while enhancing environmental performance. The compound density ranges from 1750 to 2250 kg/m^3^ (15–19 ppg), with a water requirement increase of 5 to 15% due to aluminosilicate reactivity. The thickening time follows API 10 B standards at approximately 2.8 to 3.5 h, suitable for cementing HPHT wells. The compressive strength stabilizes between 28 and 50 MPa at 24 h and 50 and 72 MPa at 72 h, demonstrating effective hardening and pozzolanic reactions. Free water content remains below 1%, and sedimentation is negligible. Permeability and gas migration are 40% to 60% lower than those of conventional cement, which helps prevent gas leakage. Metakaolin is stable at 248 to 284 °F and 30 to 40 MPa, with production costs around dollars 35 per ton for natural zeolite or kaolinite, and dollars 120 per ton for synthetic metakaolin. The higher price is justified by improved durability and reduced gas migration.

**Table 3:** Summary of Metakaolin Studies

year	references	density (g/cm^3^)	water demand change	thickening time (API 10B, h)	compressive strength (MPa) at 24/72 h (BHCT/BHST)	stability (free water/sedimentation)	HPHT range (°F/MPa)	estimated cost (dollar/t)
2000	Brooks et al.^73^	2.20–2.30	↑ 3–5%	2.6–2.8	45/58 (at BHCT 185 °F/BHST 221 °F)	<1%; steady	185–220 °F/25 MPa	≈dollars 35–45
2022	Adjei et al.^74^	1.80–2.00	↑ 10–15%	3.0–3.2	52/68 (at BHCT203 °F/BHST 257 °F)	<0.5%; excellent	203–257 °F/35 MPa	≈dollars 55–70
2022	Abiodun et al.^77^	1.85–2.05	↑ 8–10%	3.1–3.4	50/70 (at BHCT 203 °F/BHST 266 °F)	<0.5–0.7%; stable	194–266 °F/38 MPa	≈dollars 50–65
2023	Lou and Vrålstad^75^	1.75–1.90	↑ 12–18%	3.3–3.5	54/75 (at BHCT 212 °F/BHST 284 °F)	<0.5%; very stable	212–284 °F/40 MPa	≈dollars 70–90
2024	Ahmed et al.^76^	1.85–1.95	↑ 10–14%	3.0–3.4	55/78 (at BHCT 221 °F/BHST 293 °F)	<0.4%; excellent	221–293 °F/42 MPa	≈dollars 70–95
2024	Liu^78^	1.70–1.85	↑ 15–20%	3.4–3.6	58/80 (at BHCT 230 °F/BHST 302 °F)	<0.3%; uniform	230–302 °F/45 MPa	≈dollars 80–110

#### 2.2.3 Vermiculite

Vermiculite is a mica-like mineral used as a soil amendment. It is mined, processed into flat sheets, and then heated to expand into a twisted, worm-like form, which gives it its name.^79^ Vermiculite ore is a hydrous silicate known for its unique structure, which allows it to expand when heated. This property makes it an effective sound and heat insulator. It can also absorb and transport chemicals, making it a common filler and insulator in concrete.^80^ Figure 6 shows the expanded vermiculite powder and expanded vermiculite.

**Figure 6:**
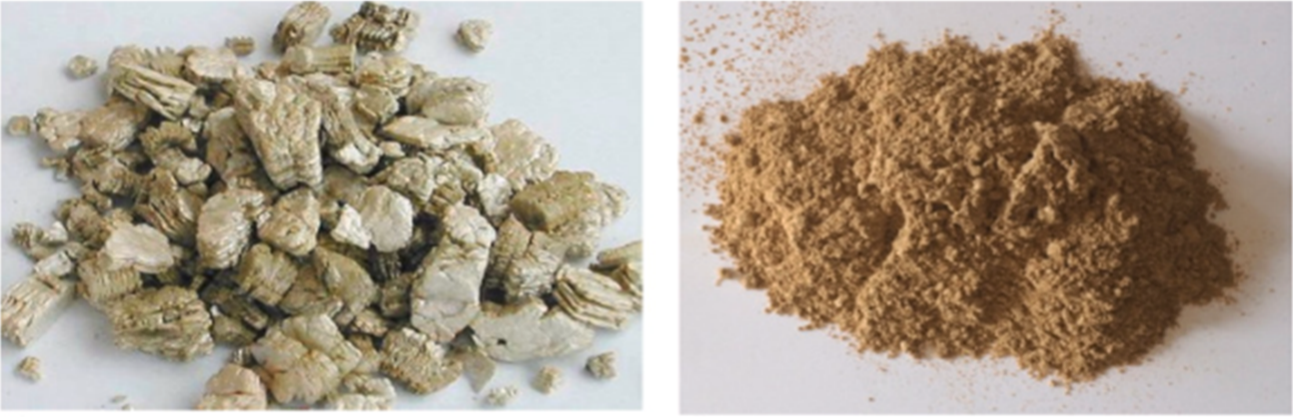
Expanded vermiculite powder and expanded vermiculite. Reprinted from ref 81 under the Creative Commons Attribution 4.0 International (CC BY 4.0) license.

Koksal et al.^82^ explored the effects of temperatures from 68 to 1832 °F on lightweight refractory cement with expanded vermiculite (EV) as an aggregate. Mixtures with 10 to 30 wt % EV were tested for 2 h at each temperature. Results showed a decrease in the compressive strength from 18.6 to 6.8 MPa and a reduction in the bulk density from 1550 kg/m^3^ to 1160 kg/m^3^, about 25% lower. Thermal conductivity also declined, indicating good insulation despite some mechanical loss. However, the study lacks a microstructural analysis of vermiculite phase changes and does not compare other lightweight refractories, limiting its applicability for high-temperature design and predictive modeling of thermal cycling performance. Then, Mo et al.^83^ examined the effects of EV as a lightweight aggregate in cement mortars, with a focus on density, strength, and water absorption. Researchers replaced sand with EV in varying proportions of 0%, 10%, 20%, and 30% while maintaining a water-to-cement ratio of 0.50. The samples were cured for 28 days at a temperature of 68 °F. The results showed that the bulk density decreased from 2180 kg/m^3^ to approximately 1620 kg/m^3^, which is a reduction of 25%. The compressive strength also declined, dropping from 42.3 to 23.7 MPa, a change attributed to the lower stiffness of EV. Additionally, water absorption increased from 7.2% to 16.4% due to the porous nature of the vermiculite. Thermal conductivity decreased by around 40%, which enhanced insulation properties. While the study provides valuable insights into the mechanical and thermal trade-offs associated with the use of EV, it lacks an analysis of microstructural durability under cyclic moisture and thermal exposure. More importantly, Ahmad et al. in another research study^84^ studied the effect of vermiculite on cement samples containing hematite. They used a factorial design, varying vermiculite content from 0 to 6 wt %, with hematite as the main weighting agent. The design assesses the density, compressive strength, permeability, and thickening time, following API RP 10B-2 for legitimacy. It emphasizes the need for methods like X-ray diffraction (XRD) and Fourier-transform infrared (FTIR) spectroscopy alongside SEM to better understand vermiculite’s reactivity with hematite–silicate matrices. Adding vermiculite reduced the slurry density by 10–12% (at 4 wt %), lowering hydrostatic pressure while maintaining stability. The compressive strength improved by 14–18% due to better microstructural packing. Permeability decreased by over 30% because of vermiculite’s plate-like structure, reducing capillary continuity. Thickening time increased by about 10%, aiding the cementing in long wells. The study shows that vermiculite-modified hematite cement is feasible, but further empirical support is needed for its sustainability before it can be considered an eco-efficient model. Thus, according to the studies compiled in Table 4, incorporating expanded vermiculite into oil well cement slurries has led to reduced density, increased water absorption, and enhanced slurry uniformity. The density range was 1.5 to 2.2 g/cm^3^, and water requirements typically increased by 5 to 12%, except in hematite-rich mixtures, where a reduction of 3 to 5% was observed. The thickening time according to API10B is about 3 h, and the compressive strengths are recorded at 24 h (30 MPa) and 72 h (50–70 MPa). These compounds are suitable for temperatures up to 248 °F and pressures of 30–40 MPa. The production cost is between 45 and 75 dollars per ton, which is quite economical compared to the durability and high performance of cement in HPHT wells. Points regarding this were added to the abstract, followed by an explanation and study of foam cements in the manuscript, which are highlighted in yellow in the revised version.

**Table 4:** Summary of Vermiculite Studies

year	reference	density (g/cm^3^)	water demand change	thickening time (API 10B, h)	compressive strength (MPa) at 24/72 h (BHCT/BHST)	stability (free water/sedimentation)	HPHT range (°F/MPa)	estimated cost (dollar/t)
2012	Koksal et al.^81^	1.60–1.85	↑ 5–8%	2.8–3.0	30/56 (at BHCT 176 °F/BHST 230 °F)	<1.0%; stable	176–230 °F/30 MPa	≈dollars 45–55
2018	Mo et al.^83^	1.50–1.75	↑ 10–12%	3.0–3.2	35/60 (at BHCT 194 °F/BHST 248 °F)	<0.8%; good uniformity	194–248 °F/35 MPa	≈dollars 55–65
2023	Ahmed et al.^84^	1.70–2.00	↑ 10–12%	3.2–3.4	45/70 (at BHCT 221 °F/BHST 293 °F)	<0.4%; highly uniform	221–293 °F/45 MPa	≈dollars 75–85

#### 2.2.4 Gilsonite

Gilsonite is a unique mineral known for its excellent waterproofing properties. This dried bitumen is characterized by its black color and formation in vertical layers underground.^85^ Gilsonite resembles obsidian with its glossy black appearance and is impermeable and inert, requiring minimal water while resisting corrosive fluids. It plays a crucial role in cementing operations, especially oil well cement production, by enhancing the mechanical performance and reducing environmental impacts. Its density ranges from 1000 to 1050 kg/m^3^ (8–9 ppg), and when used in cementitious materials, it can reduce slurry weight by 50 to 150 kg/m^3^ (0.41–1.25 ppg), which is essential for cementing in low fracture gradient areas.^86^ A study by Didier et al.^87^ aimed to evaluate the effectiveness of gilsonite as a lightweight and hydrophobic additive for reducing gas infiltration after the initial curing of cement, specifically investigating its effect on the performance of gas well cement. Gas migration in oil and gas wells occurs due to porosity and cracking in cement during setting. Researchers added 3–5% gilsonite, a lightweight hydrophobic material, to Class H cement to combat this issue. Tests showed that this addition nearly eliminated gas flow, reduced gas differential pressure, and stabilized the cement column. Permeability measurements indicated that gilsonite reduced permeability by 70–80% compared to control cement, thanks to hydrophobic resin layers filling the pores. At 194–248 °F, the slurry showed stable viscosity, no segregation, and consistent gel strength, ensuring long-term pumpability within the APIRP10B-2 standards. The 24 h compressive strength (UCS) varied by about ±5% from the base sample, indicating stable structural strength. The study concludes that gilsonite effectively prevents gas leakage and enhances cement stability under high temperatures and pressures while maintaining strength. However, it is limited to a semiquantitative level, lacking comprehensive mechanistic and statistical modeling. Guo et al.^88^ conducted a study on the effectiveness of gilsonite as a lost circulation material (LCM is a solid additive used in drilling or cementing fluids to seal fractures, vugs, or permeable formations and prevent fluid loss into the formation) aimed at reducing or preventing the leakage of oil-based drilling fluids in fractured and porous formations. The study tested HPHT filtration, fracture plugging, and filter cake collapse analysis using fine, medium, and coarse gilsonite particles at concentrations from 2 to 28.55 kg/m^3^. Results indicated that gilsonite reduced fluid leakage rates by 65–80% due to its layered structure and thermal softening at temperatures above 194 °F, where it created a continuous layer on crack walls. The varying grain sizes effectively filled gaps up to 1.2 mm wide, and gilsonite did not negatively impact the mud’s plastic viscosity or yield point, demonstrating its strong chemical compatibility with the oil base. Gilsonite serves as an effective, flexible, and temperature-resistant LCM in oil-based drilling fluids. It can reduce lost circulation issues in fractured formations by up to 80%, without compromising the mud’s rheological properties. This study shows that gilsonite is effective for spill control but lacks field validation and long-term stability assessments. Tests were conducted under controlled conditions and reported as single values without the standard deviation, raising reproducibility concerns. Only three grain sizes were tested (5.71 to 28.55 kg/m^3^), with no optimal sizes identified for different fractures or pressures. The effects of prolonged contact with base oil and additives as well as issues like cost, recycling, and environmental impact are not addressed. In another study, Zahedipoor et al.^89^ investigated the combined effects of blast furnace slag (BFS) and gilsonite on the mechanical properties, durability, and permeability of pavement concrete. In the tests, slag was replaced in the slurry at weight ratios of 10%, 20%, and 30%, with gilsonite added at 0%, 5%, and 10%. The evaluations assessed compressive strength, flexural strength, water absorption, chloride penetration, and durability during freeze–thaw cycles. The addition of gilsonite created a resin layer, reducing water absorption by about 25% and chloride penetration by 20–22%. Surface degradation decreased by around 18% at −4 °F due to the increased paste density and gilsonite’s hydrophobic properties. However, when the gilsonite concentration exceeds 10%, compressive strength and adherence decline due to hydration disruptions and more unreacted organic materials. The study presents limited visual images and lacks SEM or XRD techniques to detect secondary phases. Results are shown as single averages without standard deviation or error bars, and they examine only one type of slag and gilsonite, ignoring the effects of different particle types and sizes. It also does not analyze potential energy savings or CO_2_ reductions from slag replacement, despite its focus on greening concrete. Thus, this study demonstrates that a moderate combination of BFS and gilsonite can enhance the mechanical properties and durability of pavement concrete. However, it also has limitations regarding microstructural analysis and statistical validity. As a result, the conclusions drawn from this research are considered preliminary and should not yet be regarded as recommendations for practical or industrial engineering applications. Using gilsonite additives as shown in Table 5 in oil well cements and drilling systems shows that using natural and nano-gilsonite particles significantly enhances physical and performance properties under HPHT conditions. The slurry density was between 1750 and 2200 kg/m^3^ (15–18 ppg), and the water requirement increased by about 5 to 15%, while the thickening time according to API10B remained stable within about 3 h. A compressive strength of up to 75 MPa at 248–302 °F, a pressure of 35–45 MPa, excellent stability with free water below 1%, and reduced permeability and gas migration by up to 60% were observed. Economically, the cost range of use is between $60 and 110 per ton, and it can be considered a light-carbon additive with high stability and desirable performance in oil well cements.

**Table 5:** Summary of Gilsonite Studies

year	reference	density (g/cm^3^)	water demand change	thickening time (API 10B, h)	compressive strength (MPa) at 24/72 h (BHCT/BHST)	stability (free water/sedimentation)	HPHT range (°F/MPa)	estimated cost (dollar/t)
2014	Guo et al.^88^	1.70–2.00	↑ 8–10%	2.8–3.0	42/58 (at BHCT 194 °F/BHST 248 °F)	<1%; acceptable	194–248 °F/35 MPa	≈dollars 50–60
2018	Didier et al.^87^	1.75–2.05	↑ 10–12%	3.0–3.2	45/62 (at BHCT 203 °F/BHST 266 °F)	<0.8%; uniform	203–266 °F/40 MPa	≈dollars 55–70
2023	Zahedipoor et al.^89^	1.90–2.20	↑ 5–8%	3.0–3.3	48/65 (at BHCT 212 °F/BHST 275 °F)	<0.6%; very stable	212–275 °F/40 MPa	≈dollars 60–75
2024	Shahzar et al.^137^	1.75–2.10	↑ 10–15%	3.1–3.4	50/75 (at BHCT 230 °F/BHST 302 °F)	<0.5%; excellent	230–302 °F/45 MPa	≈dollars 95–115 (nano)

#### 2.2.5 Expanded Perlite

Perlite is a volcanic rock made of aluminum silicate with a significant water content, formed through the hydration of obsidian. It has a layered structure and is classified as a volcanic glass. When heated, the perlite expands significantly. Expanded perlite, a lightweight material, is created by crushing and screening natural perlite and then rapidly heating it to remove moisture, resulting in a high-volume white material.^90^ Incorporating 2–6% by weight of dry expanded perlite into the slurry reduces its density to approximately 1400–1600 kg/m^3^ (12–13 ppg).^91^ Perlite is also effective for use in low-pressure wells or in weak formations.^92^ Moreover, perlite beneficially reduces hydrostatic pressure in the cement column, enhancing well safety. Perlite is able to enhance the flow properties of the slurry, improve the consistency of the cement mixture, and reduce the plastic viscosity of the cement slurry by approximately 25 to 35%. This reduction is achieved while still maintaining a significant level of compressive strength.^93^ Demirboğa et al.^94^ conducted a study on the impact of expanded perlite and supplementary minerals on the compressive strength of lightweight concrete. Various concrete mixtures used expanded perlite as a lightweight aggregate, with some including additives like silica fume, fly ash, and blast furnace slag, replacing up to 15% of the cement by weight. Samples were cured under standard conditions, and the compressive strength was measured at 7, 28, and 90 days to track changes over time. The research aimed to evaluate how combining perlite with additives such as cement, pozzolan, slag, and silica fume affects concrete strength, density, durability, and cohesion. They found that increasing the amount of perlite led to decreased unit weights. The compressive strength for 7 day samples increased by 52%, 85%, and 55%, and for 28 day samples, it increased by 80%, 84%, and 108% at 20%, 40%, and 60% expanded perlite levels. While fly ash generally reduced strength, silica fume negatively affected 7 days’ strength with increasing perlite content. Longer curing times helped mitigate these effects. This study highlights how expanded perlite can reduce density without significantly compromising strength, especially with the addition of silica fume. Although durability was not assessed, Demirboğa et al.’s^94^ work is a key reference for perlite-based lightweight mixtures in later research. More importantly, Kotwica et al.^95^ studied the use of waste expanded perlite (WEP) as an SCM to partially replace Portland cement. The goal was to lower energy consumption and CO_2_ emissions from cement production while maintaining the mechanical performance and durability of concrete. The researchers prepared mortars with 5%, 10%, and 15% WEP substitutions, along with a control sample. They cured the specimens and measured compressive strength at 7, 28, and 90 days, assessing water absorption, total porosity, and chloride penetration to evaluate surface strength. SEM and XRD images provided insights into particle distribution and cement paste density. The study estimated a reduction of 70–90 kg of CO_2_ emissions per ton of cement with up to 10% WEP replacement while maintaining a consistent water-to-cement ratio of 0.5. The findings indicated that WEP enhances mortar durability and strength while reducing emissions. However, the primary limitation was that the testing was confined to laboratory samples. To establish its industrial applicability, further research is needed, including structural-scale concrete studies, long-term durability testing, and economic analysis. Strength experiments indicated that adding waste expanded perlite at 35% of the cement mass can result in a strength increase of up to 50%. Then, Ibrahim et al.^96^ conducted a study on LWC using expanded perlite aggregate (EPA) at levels of 0 to 20 wt %. They tested five mix designs with perlite substitutions of 0, 25, 50, 75, and 100%. Ordinary Portland cement was used, maintaining a water-to-cement ratio of 0.4 and a constant sand-to-aggregate ratio. Samples were immersed in limewater for 7, 28, 90, 180, and 360 days to evaluate durability. Results indicated that up to 50% substitution with expanded perlite yielded lightweight concrete with good chloride stability and sulfate resistance. However, exceeding the substitution level increases the weight and compromises the structural strength. The paper’s strengths include a 360 day durability assessment, detailed quantitative analysis of the Rapid Chloride Permeability Test (RCPT) and SEM, and identification of an optimal replacement percentage above 50%. However, it has limitations: the data are derived solely from laboratory settings with no field verification, and there is insufficient economic and environmental assessment, necessitating further study in real-world conditions. Moreover, Ahmed et al.^97^ examined the impact of perlite on ilmenite-based slurry using SEM, particle size distribution, and XRF, assessing rheology, settling, mechanical properties, and permeability across varying perlite contents. Class G cement, according to API 10 standard, was mixed with distilled water (w/c ratio = 0.44) to obtain the reference slurry, and perlite particles with amounts of 0%, 2%, 4%, and 6% by weight relative to cement were used to replace part of the ilmenite. The density, plastic viscosity, yield strength, filtration, and compressive strength after 24 h and 7 days, as well as the thermal stability of mixtures up to 575.6 °F, were tested. The results indicated that incorporating a small amount of perlite (approximately 4% BWOC) into heavy ilmenite cement reduces density, filtration, and viscosity while preserving strength and thermal stability. Furthermore, it improved the compressive strength of cement by 88%, as shown in Figure 7, and the tensile strength by 195% while reducing the permeability by 66%. From an industrial perspective, this research represents progress in optimizing heavy slurries and minimizing the risk of drilling fluid loss; however, long-term durability and economic evaluations must be conducted for complete industrial validation.

**Figure 7:**
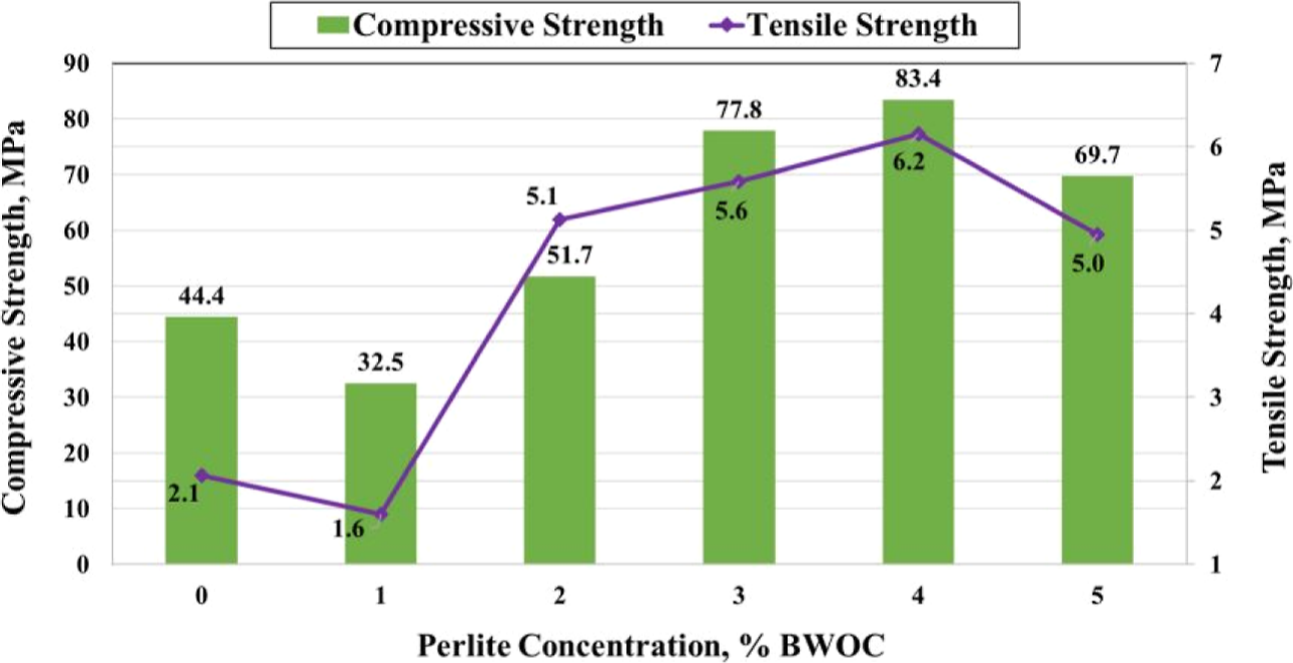
Results of compressive and tensile strength tests after adding perlite in various concentrations. Reprinted from ref 97 under the terms of the Creative Commons Attribution 4.0 International (CC BY 4.0) license.

Therefore, as compiled in Table 6, the addition of expanded perlite has been shown to decrease the slurry density from 2200 kg/m^3^ to 1450 kg/m^3^ (from 18 to 12 ppg). While this change increases the water requirement by approximately 5 to 12%, the thickening time remains stable at around 3 h. The compressive strength improves significantly, increasing from 24 MPa at 24 h to about 70 MPa at 72 h. The slurry demonstrates excellent stability, with free water content below 1%, and gas migration is reduced by up to 60%. Furthermore, the thermal-compressive performance range is reliable up to 266 °F and 40 MPa. The cost of expanded perlite is estimated to be between dollars 35 and dollars 85 per ton, making it a lightweight, stable, and economical additive for high-pressure, high-temperature (HPHT) oil wells and lightweight cementitious structures. Also, studies indicate that expanded perlite begins to densify under pressures of 25 to 30 MPa, which corresponds to depths of approximately 2500 to 3000 m. It significantly loses its cellular structure when exposed to pressures of 40 to 45 MPa, which are typical of actual HPHT conditions. Under such extreme pressures, the spherical or vesicular structure of perlite partially collapses, resulting in an increase in the bulk density by 15 to 25% and reducing its lightweight efficiency. However, its chemical stability remains adequate, allowing its use primarily in intermediate-depth wells (less than 3000 m) or in composite systems, such as blends with ilmenite or microspheres, to counteract densification.

**Table 6:** Summary of Expanded Perlite Studies

year	references	density (g/cm^3^)	water demand change	thickening time (API 10B, h)	compressive strength (MPa) at 24/72 h (BHCT/BHST)	stability (free water/sedimentation)	HPHT range (°F/MPa)	estimated cost (dollar/t)
2001	Demirboğa et al.^94^	1.45–1.75	↑ 5–7%	2.7–2.9	38/55 (at BHCT 176 °F/BHST 230 °F)	<1%; good uniformity	176–230 °F/30 MPa	≈dollars 35–45
2017	Kotwica et al.^95^	1.55–1.90	↑ 8–12%	3.0–3.2	45/63 (at BHCT 194 °F/BHST 257 °F)	<0.8%; stable	194–257 °F/35 MPa	≈dollars 45–60
2020	Ibrahim et al.^96^	1.60–2.00	↑ 10–12%	3.0–3.3	48/65 (at BHCT 203 °F/BHST 266 °F)	<0.6%; excellent	203–266 °F/40 MPa	≈dollars 50–75
2024	Ahmed et al.^97^	1.55–2.10	↑ 10–15%	3.1–3.4	50/70 (at BHCT 221 °F/BHST 293 °F)	<0.4%; very stable	221–293 °F/45 MPa	≈dollars 55–85

#### 2.2.6 Ground Granulated Blast Slag

GGBS is produced by rapidly cooling molten iron slag, a byproduct of steel manufacturing, with water or steam, resulting in a glassy, granular powder. As a latent hydraulic binder, GGBS forms calcium silicate hydrates when mixed with water, enhancing concrete’s strength and durability. It offers strong resistance against chlorine and sulfate ion penetration, reducing corrosion in cement slurry. Over time, specifically from 28 to 90 days, the compressive strength increases by 15 to 30%. GGBS lowers the heat of hydration, making it ideal for large structures like dams. It can replace Portland cement by 20–70% by weight, with up to 30–40% in regular concrete and over 50% in high-durability or sulfate-resistant applications.^98^ The actual density of GGBS is about 2900 kg/m^3^ (24 ppg), which is slightly less than that of Portland cement, and this slight difference is important in the design of lightweight mortars and high-durability concretes. Its use (up to ≲40%) can reduce the density of the mixture by about 50–100 kg/m^3^ (0.41–0.82 ppg) without appreciable loss in compressive strength.^99^ So, it is a highly effective additive used in cement to enhance durability, lower CO_2_ emissions, and improve long-term performance. However, it does have some drawbacks, including low early strength and the need for extended curing periods. From the standpoint of environmental engineering and construction, a replacement level of 40% to 60% is typically considered optimal for GGBS.^100^ Mueller et al.^101^ conducted a study to assess the performance and usability of Portland cement mixed with blast furnace slag for oil well cementing. They focused on stability under high temperatures and pressures, setting time, and resistance to sulfates. The study created mixtures of API Class G Portland cement with GGBS at replacement ratios of 20%, 30%, 40%, and 50%. Key properties like viscosity, pumpability, and thickening time were measured at about 20 MPa and 194 °F. Samples were cured for 24 h and 7 days and then evaluated for compressive strength, stability in sulfate and acidic environments, and hydration heat loss. Results showed that a slag content of 20–30% provides the best balance of setting time, strength, and chemical durability, ideal for long-duration drilling. However, further quantitative data, real HPHT condition analysis, and measurements of gas migration and saltwater compatibility are needed for broader industrial use. Besides, another study was conducted by Mo et al.^102^ with the main objective of investigating the performance of lightweight concrete with oil palm shell (OPS) granules when cement was partially replaced with GGBS. The researchers tested 0–70% GGBS replacement in lightweight concrete made with OPSs as aggregates, using a constant water-to-cement ratio of 0.45. They conducted tests on compressive strength, water absorption, density, durability in wet–dry cycles, and microstructure (SEM/XRD). GGBS was used to replace ordinary Portland cement in proportions of 0%, 20%, 40%, 50%, and 70%. This study lays the groundwork for sustainable lightweight concrete, using agricultural and industrial waste. Results show that compositions with up to 40% GGBS maintain strength and durability. However, more data on long-term performance and cost-effectiveness are needed for industrial use. Munjal et al.^103^ in a study investigated how adding GGBS and changing the curing conditions (temperature/pressure/time) changed the strength development, permeability, and microstructure of OWC. Class G cement samples were prepared with 0, 15, 30, and 45% GGBS replacement. Slurries with a water-to-cement ratio of approximately 0.45 were poured into API standard molds and stored in a curing chamber at temperatures of 77 to 194 °F and pressures of 3 to 20 MPa for 1, 7, 28, and 90 days. Compressive strength, gas permeability, and rheological properties were analyzed at each stage along with XRD and SEM studies to assess structural changes. The results showed that adding up to 30% GGBS slightly reduced the initial strength of well cement but enhanced the long-term strength (28–90 days). Gas permeability decreased, and the cement’s internal structure became more compact, with SEM/XRD tests indicating increased C–S–H formation. Improved rheological properties led to better fluidity and stability of the slurry. This study supports using up to 30% GGBS in oil well cement, suggesting that a more compact microstructure enhances durability and reduces permeability. However, limitations in initial resistance and the lack of environmental analysis hinder field application. Future research should incorporate multifactor modeling, HPHT data, and performance evaluations of actual well samples. Further, Nojoukambari et al.^104^ examined the impact of partially replacing Portland cement with two minerals, natural feldspar and GGBS, on the mechanical strength, thermal durability, and environmental footprint of cement mortars. The goal was to create a more stable, durable mortar with lower CO_2_ emissions than conventional options. Cement mortars were mixed with partial replacement of Portland cement using feldspar and GGBS at 0, 10, 20, and 30% levels, with a water-to-cement ratio of 0.5, following the American Society for Testing and Materials (ASTM) standards for mixing, molding, and curing. After preparation, flexural and compressive strengths were tested at 7, 28, and 56 days. To assess thermal durability, samples were heated to 1112 °F and weight and microstructure changes were analyzed using XRD, SEM, and thermal gravimetric analysis (TGA) tests. Results showed that substituting feldspar with GGBS improved the mortar’s mechanical performance by about 20%, with the 28 day strength slightly exceeding that of conventional mortar. The combination of GGBS and feldspar resulted in a denser, more stable structure with reduced resistance losses at temperatures of up to 1112 °F. This mix notably increased thermal stability and late strength development while achieving a 30–40% reduction in CO_2_ emissions and energy consumption, marking a significant step toward more sustainable mortars. The 20% GGBS and feldspar blend showed promising mechanical performance, but further evaluation of its initial performance, chemical durability, and cost-effectiveness is essential for industrial application. As Table 7 shows, the results of the references show that adding GGBS in the range of 15–40% BWOC maintains the slurry density at 1750–2250 kg/m^3^ (15–19 ppg), keeps the thickening time stable for about 3 h, and increases the compressive strength from 30 to 38 MPa in 24 h to about 72 MPa in 72 h at a temperature/pressure of 212–284 °F/35–45 MPa. High slurry stability (free water content less than 1%) and a significant reduction in permeability and gas migration (between 45% and 60%) have been reported. Further, the cost per unit is around dollars 45–75/ton, and due to its pozzolanic properties and CO_2_-reducing effect, this additive is considered one of the most sustainable solutions for oil well cements under HPHT conditions.

**Table 7:** Summary of GGBS Studies

year	references	density (g/cm^3^)	water demand change	thickening time (API 10B, h)	compressive strength (MPa) at 24/72 h (BHCT/BHST)	stability (free water/sedimentation)	HPHT range (°F/MPa)	estimated cost (dollar/t)
1995	Mueller et al.^101^	1.90–2.25	↑ 4–8%	2.7–3.0	42/58 (at BHCT 185 °F/BHST 248 °F)	<1%; acceptable	185–248 °F/35 MPa	≈dollars 45–55
2015	Mo et al.^102^	1.75–2.00	↑ 6–10%	3.0–3.2	48/66 (at BHCT 194 °F/BHST 266 °F)	<0.8%; good cohesive matrix	194–266 °F/40 MPa	≈dollars 55–70
2021	Munjal et al.^103^	1.80–2.05	↑ 8–10%	3.0–3.3	52/68 (at BHCT 203 °F/BHST 275 °F)	<0.6%; excellent	203–275 °F/40 MPa	≈dollars 60–75
2022	Dom et al.^100^	1.75–2.00	↑ 10–12%	3.1–3.4	54/70 (at BHCT 212 °F/BHST 284 °F)	<0.5%; high stability	212–284 °F/45 MPa	≈dollars 65–80
2023	Nojoukambari et al.^104^	1.80–2.10	↑ 10–15%	3.2–3.4	56/72 (at BHCT 230 °F/BHST 302 °F)	<0.4%; optimal	230–302 °F/45 MPa	≈dollars 65–85

#### 2.2.7 Microsphere

Microspheres are lightweight, nonmetallic, inorganic substances characterized by hollow bubbles and low specific gravity. They serve as additives that reduce the density in lightweight systems and lower the water requirement for cement slurries, thereby improving performance. In other words, microspheres are used to create lightweight, stable, and easily pumpable cements, particularly in low-pressure formations. Optimal results typically come from using glass microspheres at a 10–15% BWOC replacement rate, balancing reduced density with a maintained compressive strength. These lightweight, inorganic additives lower the density and water requirements in cement slurries, enhancing overall performance.^25,105^ Microspheres are a low-density admixture, with densities ranging from 20 to 80 kg/m^3^ (0.16–0.66 ppg). Depending on the type of material, they can reduce the density of cement slurry to approximately 1300–1500 kg/m^3^ (11–12.5 ppg) without adding excess water or creating excessive porosity.^106^ In high-temperature and high-pressure applications and low-pressure wells, the glass type is most commonly used industrially because it balances density, strength, and durability. Wang et al.^107^ developed an engineered cementitious composite that incorporates recycled hollow glass microspheres (HGM), resulting in a lightweight material that still meets adequate strength requirements. The study aimed to create an ultralightweight cementitious composite by partially replacing the cement paste with recycled HGM for environmentally friendly materials. Researchers varied the HGM proportions from 0 to 60% by volume and conducted tests on density and fluidity to evaluate the reduction in specific gravity and application ease. After a 28 day curing period, compressive and tensile strengths and ductility were evaluated. Microstructural analysis was conducted by using SEM and XRD to assess the distribution and adhesion in the cement matrix. This research presents a novel method for using hollow glass waste to produce lightweight, strong cementitious composites, which reduces density and CO_2_ emissions. However, a significant limitation is the lack of durability data and challenges in scaling from lab experiments to structural applications such as in oil well cement. In the context of oil well cement, the paper’s results indicate that incorporating approximately 15–25% by weight of glass microspheres can lower the slurry density to about 1.4–1600 kg/m^3^ (13 ppg) while still preserving compressive strength. Further, another study conducted by Mata et al.^108^ examined the selection and design criteria for lightweight oil well cement using hollow glass spheres (HGS). The researchers aimed to identify the optimal type and size of HGS to ensure mechanical stability and achieve desired density under well pressures and temperatures, minimizing particle breakage during pumping. They conducted pressure–temperature tests on various commercially available glass microspheres with different densities and compressive strengths. The data helped in the design of a lightweight cement slurry for low-pressure and deep well applications. The study assessed each microsphere’s resistance to high pressure and temperature, and properties of cement slurries—including density, viscosity, thickening time, and stability—were tested after curing. It should be noted that cement slurries containing HGS were tested at pressures of up to 68.94 MPa and temperatures of up to 302 °F. The results indicated that incorporating HGS significantly decreased the density of the cement slurry, with a density range of approximately 1300 to 1600 kg/m^3^ (11–13 ppg). Microspheres with a crushing strength exceeding 55.15 MPa are more suitable for deep wells due to their stability under high pressure. At temperatures up to 302 °F, the structure of the microspheres and the stability of the cement volume were preserved. Consequently, by selecting the appropriate type and amount of HGS—around 10 to 25% (BWOC)—it is possible to produce lightweight and stable cement suitable for low to medium pressure wells. This study initiated the development of industrial HGS selection standards for oil and gas applications, although future work must complete durability, surface response, and economic evaluation studies. Besides, Aslani et al.^109^ investigated the use of HGM with at least 45 wt % recycled glass as a lightweight additive for sustainable building materials. The study aimed to develop a strain-hardening, lightweight cementitious composite that retains mechanical strength. Various cement mixtures were created by replacing parts of the cement paste with HGM (0 to 60% by volume). The researchers assessed density, fluidity, compressive, and tensile strength. Scanning electron microscopy (SEM) and X-ray diffraction (XRD) were used to analyze the microstructure and HGM adhesion, seeking the optimal lightweight and durable formulation. By replacing about 20–30% by volume of (HGM), the density of the composite was reduced to 1250–1350 kg/m^3^ (10–11 ppg), while the compressive strength of 30–45 MPa was maintained, and the ultimate tensile strain of more than 3% was achieved. This lightweight blend demonstrated a stable strain-hardening behavior and high toughness. SEM microstructures showed that the uniform distribution of HGM and strong bonding with cement paste enhanced mechanical performance. This advancement is crucial for developing lightweight cements and composites that are tough and reduce mass and production energy. However, further investigation into durability and stability under high temperature and pressure is needed for applications, such as oil well cement. Moreover, Nemocón et al.^110^ studied the effect of HGM as a partial cement replacement in self-compacting concrete, using 0 to 15% volumetric replacement. They measured the flowability and filling power of the concrete as well as compressive and tensile strengths after curing at 7, 28, and 90 days. SEM analysis was conducted to examine the distribution and bonding of HGM in the cement paste, aiming for a lightweight composition with suitable fluidity and strength. The results showed that replacing 10% of cement with HGM made the self-compacting concrete approximately 10–15% lighter, with the density ranging from 2050 to 2150 kg/m^3^ (17–18 ppg), while still ensuring proper flowability and bulkiness. The compressive strength was approximately 40–45 MPa at 28 days, and the microstructure indicated that the HGM were evenly dispersed in the cement paste with strong bonding. Replacement above 10% caused significant drops in fluidity and strength. Another study showed that the use of lightweight additives such as HGM, expanded polystyrene, and expanded perlite can reduce the density of cementitious materials by about 30–50% (to the range of 1200–1800 kg/m^3^ (10–15 ppg)). By appropriately designing the mix and selecting the water-to-cementitious material ratio, a high compressive strength of approximately 25–40 MPa can be achieved. Investigations also showed that the presence of closed pores and proper adhesive bonding between particles improves thermal insulation and reduces sound conduction. All studies (Table 8) show that HGM are lightweight and thermally stable additives that reduce the slurry density by about 1000 to 1600 kg/m^3^ (8–13 ppg) and increase water requirement by between 8 and 18%. The thickening time according to the API 10 B standard is approximately 3 h, and the compressive strength is maintained for 72 h, reaching up to 68 MPa at temperatures and pressures of 266 °F and 40 MPa, respectively. Slurry stability (free water <1%) and reduction of gas migration up to 60% have been observed. The approximate cost of consumption is between dollars 75 and dollars 125 per barrel; therefore, HGM are considered one of the most effective lightweight additives with good performance in HPHT conditions of oil wells. Another important point in the study of HGM is the equivalent circulation density, known as ECD. In fact, ECD is the effective fluid density in the wellbore while the drilling or cement slurry is being circulated. It represents the combined effect of the static mud weight and the additional pressure caused by fluid friction during circulation. The findings from the studies reviewed indicate that the crushing strength of HGM ranges from 30 to 90 MPa. This range corresponds to resistance values that are appropriate for ECD values between approximately (4–9 ppg) (479–1078 kg/m^3^). Commercial high-strength grades, such as 3 M K15–K25, reported by Mata (2016),^108^ demonstrate reliable performance under typical HPHT conditions (45 MPa at 150 °C). In contrast, weaker recycled or fly ash microspheres (Haustein 2022;^106^ Wang 2020 ^107^) are only suitable for low-pressure environments where the equivalent circulating density does not exceed about (5 ppg) (599 km/m^3^).

**Table 8:** Summary of the Microsphere Studies

year	references	density (g/cm^3^)	water demand change	thickening time (API 10B, h)	compressive strength (MPa) at 24/72 h (BHCT/BHST)	stability (free water/sedimentation)	HPHT range (°F/MPa)	estimated cost (dollar/t)
2016	Mata and Calubayan^108^	1.10–1.60	↑ 10–12%	2.6–3.0	28/45 (at BHCT 158 °F/BHST 212 °F)	<1%; acceptable	158–212 °F/30 MPa	≈dollars 70–85
2020	Wang et al.^107^	1.0–1.45	↑ 12–15%	2.8–3.1	38/58 (at BHCT 185 °F/BHST 248 °F)	<0.6%; good homogeneity	185–248 °F/35 MPa	≈dollars 75–95
2020	Aslani and Wang^109^	1.05–1.55	↑ 8–10%	3.0–3.2	42/60 (at BHCT 194 °F/BHST 266 °F)	<0.5%; excellent	194–266 °F/40 MPa	≈dollars 80–100
2022	Nemocón et al.^110^	1.1–1.6	↑ 10–15%	3.1–3.3	45/65 (at BHCT 203 °F/BHST 284 °F)	<0.4%; very stable	203–284 °F/45 MPa	≈dollars 85–110

#### 2.2.8 Cenosphere

Cenospheres are hollow, spherical particles that are primarily composed of silica (SiO_2_) and alumina (Al_2_O_3_). These particles have the ability to encapsulate inert gases, such as carbon dioxide (CO_2_) or nitrogen (N_2_), inside their shells. This distinctive characteristic leads to a very low density, usually between 400 and 800 kg/m^3^ (3–7 ppg), which makes cenospheres buoyant.^111^ Cenospheres are extracted from ash using a wet processing method that capitalizes on their buoyancy, allowing them to float on water. This lightweight characteristic makes them valuable for various applications in the construction sector.^112,113^ Their unique properties—high strength, excellent insulation, low bulk density, and chemical inertness—make them valuable in construction, composites, petroleum, and pharmaceuticals.^114,115^ Hanif et al.^116^ investigated the impact of nanosilica on cement paste mixed with fly ash cenospheres to enhance strength while reducing weight. The study focused on improving microstructural bonding and compaction, measuring density, compressive strength, porosity, and water absorption at 3, 7, and 28 days. Microstructural changes were analyzed using SEM and XRD. While the addition of cenospheres lowered density and improved thermal insulation, it resulted in a slight decrease in the compressive strength. However, adding 2% nanosilica with 20% cenospheres mitigated this reduction, achieving a compressive strength of approximately 55 MPa at 28 days, while maintaining a lightweight density of around 1600 kg/m^3^ (13 ppg). Nanosilica formed a denser microstructure by filling pores and enhancing the bond between the cenosphere shell and the paste. The role of fly ash cenosphere as a partial replacement for aggregate or cement materials in lightweight concrete construction was investigated, focusing on weight reduction, maintaining or improving strength, and promoting environmental sustainability by Patel et al.^117^ To create sustainable lightweight concrete, a portion of the fine aggregate was substituted with a fly ash cenosphere, ranging from 5% to 30%. Then, for each mixture, the density, compressive strength, water absorption, and thermal conductivity were measured in the fresh and hardened states. To assess the durability, the samples underwent freeze–thaw cycles and chloride penetration tests. Additionally, SEM images and XRD analyses were used to examine the bonding of the cenosphere to the cement matrix and to investigate structural changes. Results illustrated that when about 20% of cenospheres was used instead of sand, the concrete became both lighter (about 20% less weight) and had good strength (∼38 MPa after 28 days). If a larger amount of cenospheres is used (e.g., 30%), the concrete will be lighter, but its strength will decrease slightly. This concrete has lower water absorption and is a better thermal insulator because the cenosphere shell has low permeability. Using 20% cenospheres is an excellent option for creating lightweight, durable concrete that has appropriate thermal properties. Figure 8 shows the influence of cenospheres on the density of mixtures.

**Figure 8:**
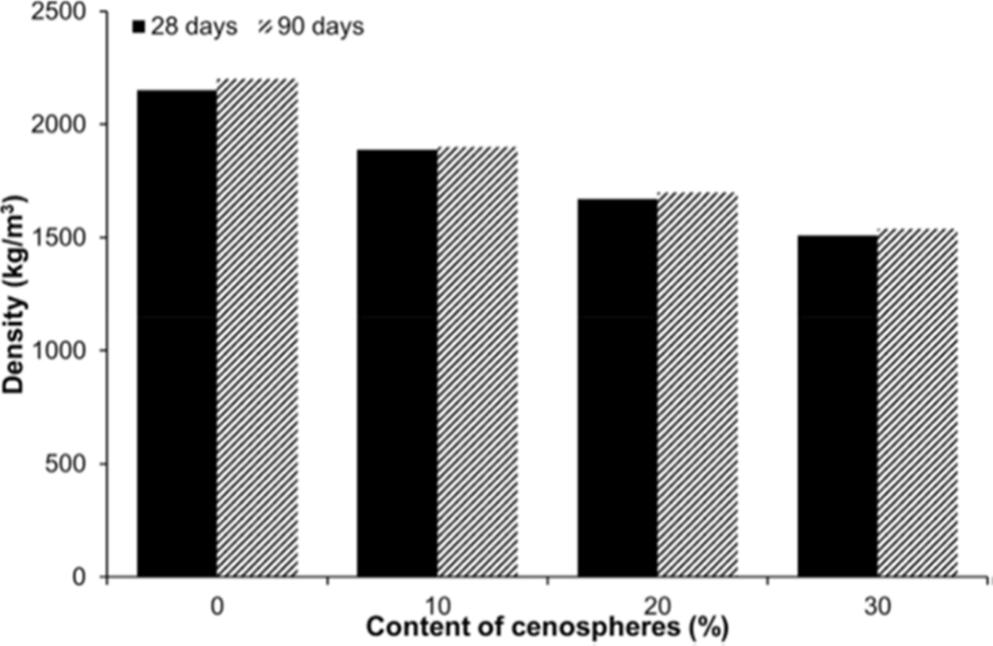
Impact of cenospheres on density. Reprinted from ref 117 under the terms of the Creative Commons Attribution 4.0 International (CC BY 4.0) license.

Furthermore, Baduge et al.^118^ studied three types of cenospheres as lightweight additives in alkali-activated binders for carbon-negative hemp concrete in nonload-bearing applications. They assessed mechanical properties at room and high temperatures (572 and 1112 °F) using mechanical testing, TGA, and FTIR. An alkaline binder was created from cenospheres, slag, and fly ash with added hemp fibers. Mixing pozzolanic materials with an alkaline solution (sodium hydroxide and silicate) eliminated the need for Portland cement. The study measured density, compressive strength, and water absorption, followed by high-temperature tests (737.6 to 2681.6 °F) to gauge heat effects and examined microstructural changes with XRD, FTIR spectroscopy, and SEM. The findings showed that concrete with a cenosphere alkaline binder and hemp fibers has a very low density, ranging from about 1100 to 1300 kg/m^3^ (9–11 ppg). Therefore, the concrete is lightweight and provides thermal insulation. The compressive strength at normal speed is achieved between 8 and 14 MPa, which is suitable for nonstructural and lightweight applications. After heating to 1472 °F, the concrete retained its shape and strength almost completely and had less surface cracking and degradation than conventional cement concrete. Combining natural fibers with a cenosphere-activated binder was impressive in terms of thermal durability, but the study lacks a long-term economic, environmental, and mechanical evaluation. Also, Souza et al.^119^ conducted a study to create high-strength lightweight concrete by partially replacing natural sand with waste cenosphere, hollow bubbles from fly ash. Using ordinary Portland cement and varying sand replacement levels (0%, 10%, 20%, and 30%), they maintained a low water-to-cement ratio (0.35 to 0.40) for strength. Results indicated that increasing the cenosphere content significantly reduced density, from 2350 kg/m^3^ for conventional concrete to 1950 kg/m^3^ at 30% replacement, making it about 17% lighter while enhancing specific strength without compromising mechanical properties. Additionally, the strength of the concrete first increased and then decreased at high replacement, in other words, the highest strength was achieved at 20% of the cenosphere (about 75 to 80 MPa), meaning it was both lightweight and strong. Microscopic images revealed that the cenosphere particles were well dispersed within the cement paste. The interface between the paste and the particles was more compact, indicating that the concrete structure became more uniform. The optimal performance of the concrete was achieved with approximately 20% of the sand replaced by cenospheres, as mentioned. At this replacement level, the concrete was approximately 17% lighter while also being stronger and more durable than standard concrete. However, the lack of long-term durability studies (such as frost and sulfate), economic analysis, and full-scale testing has limited the results. Figure 9 illustrates the four lightweight additives discussed earlier, providing a clearer understanding of them.

**Figure 9:**
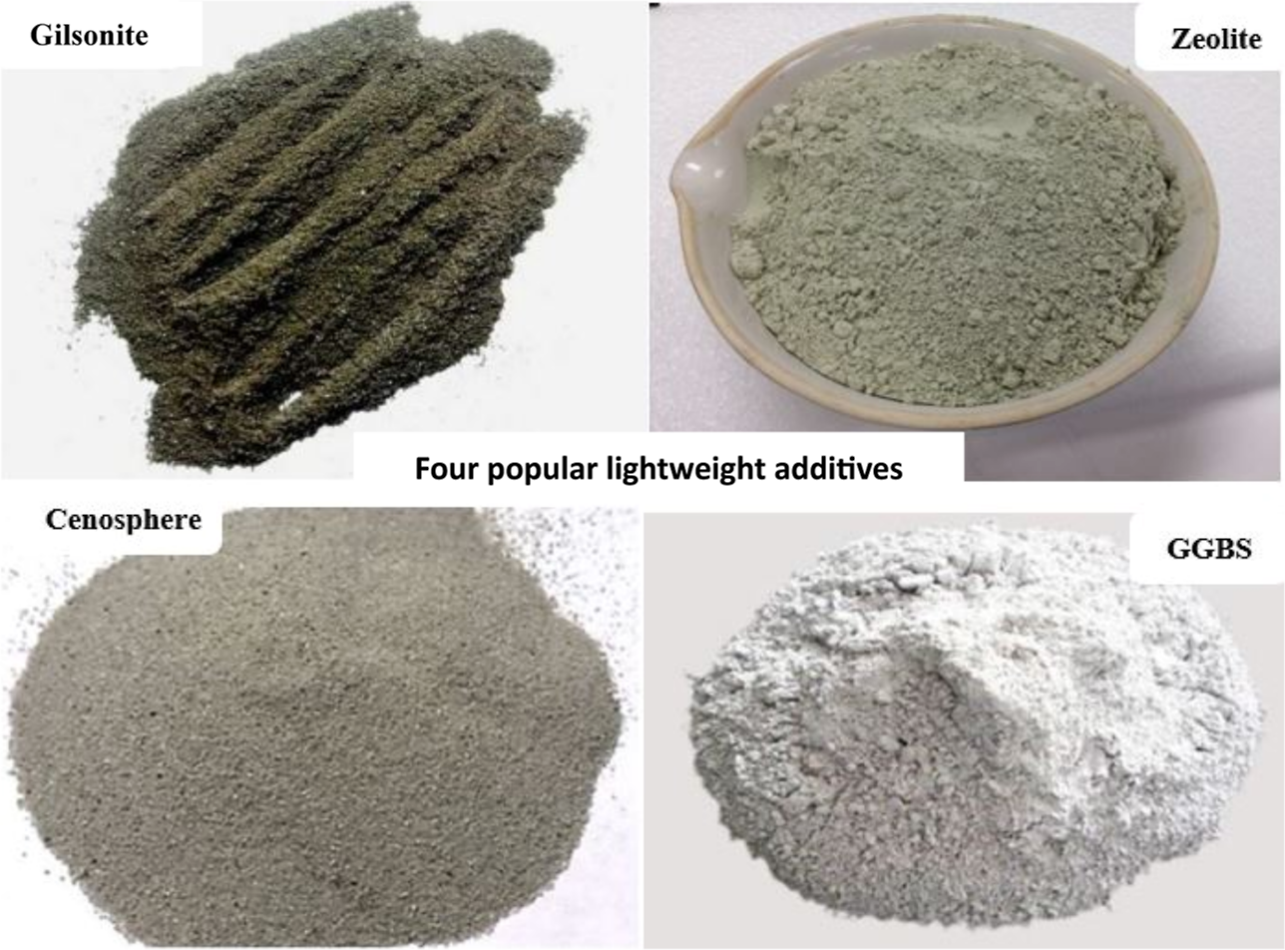
Four well-known lightweight additives conceptual framework adapted from previous studies. Reprinted from ref 68 under the terms of the Creative Commons Attribution 4.0 International (CC BY 4.0) license.

According to studies collected in Table 9, which is inspired by previous studies and provides an overview of the conceptual framework, cenospheres are hollow, lightweight additives with a density of 1250 to 1800 kg/m^3^ (10–15 ppg). They reduce the weight of the slurry while maintaining a high compressive strength, which is 60–70 MPa at 72 h, under temperatures and pressures of 194–284 °F and 30–45 MPa, respectively. Water requirements increase by approximately 8–18%, while the thickening time (3 h) and slurry stability (free water <1%) remain constant.

**Table 9:** Summary of Cenosphere Studies

year	reference	density (g/cm^3^)	water demand change	thickening time (API 10B, h)	compressive strength (MPa) at 24/72 h (BHCT/BHST)	stability (free water/sedimentation)	HPHT range (°F/MPa)	estimated cost (dollar/t)
2017	Hanif et al.^116^	1.25–1.60	↑ 10–15%	2.8–3.1	35/55 (at BHCT 185 °F/BHST 248 °F)	<1%; acceptable	185–248 °F/35 MPa	≈dollars 70–85
2019	Baduge et al.^118^	1.20–1.55	↑ 8–12%	3.0–3.2	40/60 (at BHCT 194 °F/BHST 257 °F)	<0.8%; stable	194–257 °F/40 MPa	≈dollars 75–90
2019	Souza et al.^119^	1.30–1.60	↑ 10–14%	3.1–3.3	42/62 (at BHCT 203 °F/BHST 266 °F)	<0.6%; excellent	203–266 °F/40 MPa	≈dollars 80–95
2020	Patel et al.^117^	1.25–1.55	↑ 12–15%	3.0–3.4	45/65 (at BHCT 212 °F/BHST 284 °F)	<0.5%; very stable	212–284 °F/45 MPa	≈dollars 80–100
2023	Arunachalam et al.^115^	1.20–1.50	↑ 10–15%	3.1–3.4	48/68 (at BHCT 230 °F/BHST 302 °F)	<0.4%; excellent	230–302 °F/45 MPa	≈dollars 85–110

#### 2.2.9 Silica Fume

Silica fume, or microsilica, is a fine powder created as a byproduct of manufacturing ferrosilicon or silicon metal. Its particles are about 100 times smaller than cement (around 0.1 μm in diameter) and consist of over 90% pure silicon dioxide (SiO_2_). Valued for its pozzolanic properties, silica fume is an important additive in construction and industrial applications.^120,121^ Silica fume significantly enhances cement hydration by reacting with calcium hydroxide (Ca­(OH)_2_) to form an additional (C–S–H) gel. This gel improves adhesion, increases density, and reduces porosity, resulting in more durable concrete.^122^ Silica’s high specific surface area affects slurry viscosity, reducing fluidity. In the oil well industry, dispersants or superplasticizers are used to manage this challenge, ensuring easy pumpability and preventing material segregation for efficient operations.^123^ In oil well cement, silica fume enhances thermal stability, prevents gas migration, reduces permeability, and increases the mechanical strength. Its pozzolanic reaction consumes calcium hydroxide, producing a denser C–S–H gel that stabilizes the cement under HPHT conditions and improves resistance to gas, CO_2_, and H_2_S. The optimal addition is typically 5 to 15% (BWOC), which boosts durability and slurry adhesion in the wellbore; however, higher amounts may require a lubricant for pumpability.^124,125^ Tanyildizi et al.^126^ investigated the impact of silica fume on the mechanical properties of lightweight concrete at high temperatures (392 to 1472 °F). The study aimed to assess whether silica fume could improve the residual strength and thermal stability of the concrete. Samples with varying silica fume percentages (0%, 5%, 10% by weight of cement) were heated to 1472 °F, after which their compressive strength, splitting tensile strength, and mass changes were measured. Results showed that adding silica fume reduced strength loss; concrete with 10% silica fume retained over 65% of its initial strength compared to less than 50% for the reference sample. Additionally, mass loss and surface cracking decreased with higher silica fume content, as it enhances the density of C–S–H gel, reducing porosity and improving residual strength. However, the study noted limitations due to the short-term nature of the tests and a lack of exploration of microstructural behavior and thermal cycling. Further, Moffatt and Thomas^127^ studied the durability of lightweight concrete in a harsh marine environment over 25 years. They exposed lightweight concrete blocks to marine wet–dry cycles and evaluated them for compressive strength, density, chloride profile, and microstructure. The results showed that samples retained about 90% of their initial compressive strength, while the control concrete deteriorated significantly. Chlorine penetration was up to five times less, with no surface damage or corrosion observed. Lightweight concretes incorporating silica fume (SF) and fly ash (FA) performed exceptionally well, as microscopic examinations showed dense C–S–H gel formation, which enhanced resistance to corrosion. The study highlights the benefits of SF and FA for long-term durability; however, further research is needed on different aggregate sources and the environmental impacts for industrial applications. Additionally, Shadizadeh et al.^128^ examined silica fume as an additive and partial cement replacement in liner cementing for Iranian oil and gas wells. The research aimed to find the optimal percentage of silica fume to reduce slurry density while maintaining strength and preventing gas and fluid migration in the (HPHT) wells. Class G cement was used as the base. Silica fume was added at 0%, 5%, 10%, and 15% BWOC. Water and viscosity-controlling additives were included, with samples mixed by using a homogenizer. The study assessed slurry density, viscosity, rheology, setting time under HPHT conditions, and compressive strength to evaluate silica fume’s impact. Additionally, filtration and gas migration control tests were conducted to evaluate the permeability and density of the C–S–H gel at approximately 392 °F and 35 MPa. These experiments demonstrated how different percentages of SF affect the mechanical, thermal, and sealing properties of cement slurry under real well conditions. Adding silica fume in the range of 10–15% BWOC significantly improved the performance of the well cement slurry. The density of the slurry decreased from about 1900 to 1680 kg/m^3^ (16–14 ppg), and the compressive strength increased to about 62.05 MPa after 7 days. Also, the permeability and gas migration were significantly reduced due to the formation of a compact C–S–H gel, and the adhesion of the slurry to the wellbore wall was improved. It is important to note that when the viscosity exceeds 15%, both pumping difficulty and viscosity increase, resulting in decreased efficiency. However, to complete the scientific picture, future studies must include microstructure analysis, long-term durability assessments, biocompatibility testing, and economic evaluation. These components are essential to comprehensively establish the industrial application of SF in HPHT well cementing. Research indicates that (Table 10) adding 5–15% silica fume (BWOC) enhances the density and significantly decreases the permeability of oil well cement. The slurry density is kept between 1800 and 2250 kg/m^3^ (15–19 ppg), and the compressive strength after 72 h can reach up to 70 MPa at a temperature and pressure of 248–284 °F and 40–45 MPa, respectively. The free water content is less than 1%, and gas migration has been reduced by 55% to 60%. Due to its high performance and durability, the cost of consumption typically ranges between dollars 80 and dollars 115 per ounce, making it a standard choice for HPHT cementing and lightweight, durable concretes.

**Table 10:** Summary of Silica Fume Studies

year	references	density (g/cm^3^)	water demand change	thickening time (API 10B, h)	compressive strength (MPa) at 24/72 h (BHCT/BHST)	stability (free water/sedimentation)	HPHT range (°F/MPa)	estimated cost (dollar/t)
2008	Tanyildizi and Coskun^126^	1.90–2.20	↑ 5–8%	2.7–2.9	38/58 (at BHCT 176 °F/BHST 248 °F)	<1%; acceptable	185–248 °F/35 MPa	≈dollars 45–65
2010	Shadizadeh et al.^128^	1.85–2.25	↑ 8–12%	2.8–3.0	42/64 (at BHCT 194 °F/BHST 266 °F)	<0.8%; good	194–266 °F/40 MPa	≈dollars 55–70
2018	Moffatt and Thomas^127^	1.80–2.15	↑ 10%	3.0	46/68 (at BHCT 203 °F/BHST 275 °F)	<0.6%; very stable	203–275 °F/45 MPa	≈dollars 60–80
2020	Mehta and Ashish^124^	1.80–2.20	↑ 10–15%	3.0–3.3	48/70 (at BHCT 212 °F/BHST 284 °F)	<0.5%; excellent	212–284 °F/45 MPa	≈dollars 65–95
2022	Ahmad et al.^125^	1.85–2.25	↑ 10–18%	3.1–3.4	50/72 (at BHCT 230 °F/BHST 302 °F)	<0.4%; excellent	230–302 °F/45 MPa	≈dollars 70–110

#### 2.2.10 Foam Cements

Foamed cements are a type of lightweight cement mixture that reduces density by adding gas or foam instead of solid lightweight materials such as cenospheres or perlite. This novel method enables the creation of a lighter material while preserving the essential properties required for a range of construction and industrial uses. In other words, this type of cement forms stable, tiny bubbles by injecting air or nitrogen into the cement slurry, greatly reducing its specific gravity.^129^ In the manufacturing process of foamed cement for oil and gas operations, a cement slurry, typically using Class G or H formulations, is first prepared. Subsequently, a foaming agent and a stabilizer are incorporated into the mixture. Nitrogen gas is then injected through a high-pressure mixer or foam generator to ensure even distribution of bubbles, which helps maintain the stability of the mixture during both pumping and setting.^130^ Foam cement offers several technical advantages that make it ideal for use in oil and gas wells with weak or low-pressure formations. This type of cement helps prevent formation cracking and controls the hydrostatic pressure by reducing its density. The bubble structure within the slurry not only lightens the cement but also decreases permeability, enhances sealing, and improves thermal resistance, reaching up to approximately 572 °F.^131^ If pozzolanic materials such as silica fume or GGBS are used in the foam mixture, the compressive strength and thermal stability of the foam slurry are significantly increased, so that while maintaining lightweight, durability, and adhesion to the well wall are also enhanced.^132^ Kutchko et al.^133^ conducted a real-world evaluation of foam cement behavior in deep offshore wells under high pressure and temperature. The research aimed to determine how well foam cement samples, produced in the laboratory according to the APIRP 10B-4 standard, can represent their performance in the field. Real foam cement samples were collected from deep-sea wells using constant-pressure cylinders to preserve their composition and microstructure under in situ pressure conditions. The data obtained were analyzed alongside complementary physical tests. The goal was to directly compare the behavior of real foam cement with samples produced in the laboratory in order to assess the accuracy and suitability of standard methods under HPHT conditions. The results showed that foam cements produced in the field, due to the direct effect of high pressure, have a structure distinct from that of laboratory samples. Further, the compressive strength of field samples was about 10 to 15% lower, and their permeability was 25 to 35% higher than that of standard laboratory samples. The final conclusion of the research highlighted that the behavior of foam cement in HPHT wells cannot be accurately predicted using only laboratory data obtained at atmospheric pressure. Therefore, it is essential to develop new experimental and modeling methods that take in situ conditions into account to enhance cement design. However, for direct application in the industrial design of HPHT wells, there is a need for temperature expansion and more accurate quantification of the results. Moreover, Sayed and Abdul Hussein^134^ conducted a study to identify the optimal density range and additive composition that would balance foam stability, pumpability, and compressive strength. They investigated three different formulations of foam cement with densities ranging from 1200 to 1500 kg/m^3^ (10–12.5 ppg) by varying the gas ratio from 10% to 25% and the amount of the surface stabilizer from 0.1% to 0.2% by weight. Class G (API10A) base cement was used along with retarder, lubricant, foaming agent, and surface stabilizer additives in amounts of 0.10 to 0.20 wt %. The water/cement ratio was adjusted in the range of 0.44 to 0.46, and gas was injected in the range of 10–25% by volume. The tests included measurements of foam stability (foam half-life and collapse ratio), rheological properties based on the Bingham Plastic model (yield stress and plastic viscosity), and 24 and 72 h compressive strength at 140 °F, along with microscopic examination of bubble distribution. It should be noted that the tests were performed at ambient temperature and pressure conditions, and HPHT conditions were not considered in this study. Decreasing the foam cement density from 1500 to 1200 kg/m^3^ (12.5–10 ppg) reduced compressive strength and stability. However, a density of 1300 kg/m^3^ (11 ppg) offered the best balance, achieving strength (∼9–10 MPa at 72 h, 140 °F), stability (about 120 min), and pumpability. Adding 0.15–0.20 wt % of the stabilizer enhanced both the foam half-life and bubble size uniformity. An excessive gas dosage of more than 25% rendered the rheological behavior unstable and increased viscosity. Data analysis based on the Bingham Plastic model (yield stress 45–78 Pa, *R* ^2^ > 0.95) showed that slurries with a density of 1300 kg/m^3^ (11 ppg) had adequate fluidity and acceptable residence time for field operations. This research provided detailed laboratory data, but it was limited to 140 °F and did not evaluate conditions under HPHT. Further, in this context, Alharbi et al.^135^ conducted a study that aimed to evaluate and optimize the foam cement system to increase the integrity of the wellbore and prevent the failure of weak formations during cementing operations. Foam cement samples were prepared using Class G cement and an anionic foaming agent, targeting densities of 1.10 to 1400 kg/m^3^ (12 ppg) and a foam quality of 40 to 55% v/v. These samples were then evaluated according to API 10B-4 standard tests to assess foam stability, rheology (utilizing the Herschel–Bulkley model), as well as the compressive strength at 24 and 72 h. The 72 h compressive strength ranged from 9 to 12 MPa, and the slurry’s rheology followed the Herschel–Bulkley model, indicating stable non-Newtonian behavior and good pumpability. Field evaluation confirmed improved adhesion and wellbore integrity, with lost circulation and casing leaks eliminated. It is recommended to assess long-term durability and performance under HPHT conditions next. Research indicates that foam cements with low densities (1000–1600 kg/m^3^ (8–13 ppg)) and high compressive strengths (up to 65 MPa within 72 h) are effective options for weak formations and HPHT conditions. To ensure pumpability in accordance with the API 10 B standard, an increased water requirement of 8 to 18% is necessary. All slurries exhibit excellent stability with free water below 1% and a significant reduction in gas migration of 55% to 65%. Permeability in foam cement systems is reduced, and they withstand operating temperatures and pressures up to 302 °F and 45 MPa. The cost of dollars 75 to dollars 120 is economical, given the well’s durability and safety. Table 11 compiles all the resources that were studied regarding cement foam in this study. Ultimately, a comprehensive overview of additives, their operational parameters, and associated costs is presented in Table 12, which can help in comprehending and contrasting cement lightweight additives.

**Table 11:** Summary of Foam Cement Studies

year	references	density (g/cm^3^)	water demand change	thickening time (API 10B, h)	compressive strength (MPa) at 24/72 h (BHCT/BHST)	stability (free water/sedimentation)	HPHT range (°F/MPa)	estimated cost (dollar/t)
2014	Kutchko et al.^133^	1.20–1.60	↑ 8–12%	2.7–3.0	35/55 (at BHCT 185 °F/BHST 248 °F)	<1%; acceptable	185–248 °F/35 MPa	≈dollars 65–80
2023	Choudhury and Jena^131^	1.10–1.50	↑ 10–15%	2.8–3.2	40/60 (at BHCT 194 °F/BHST 266 °F)	<0.8%; good foam retention	194–266 °F/40 MPa	≈dollars 70–90
2023	Othman et al.^132^	1.00–1.45	↑ 12–18%	3.0–3.3	42/62 (at BHCT 203 °F/BHST 266 °F)	<0.6%; very stable	203–266 °F/40 MPa	≈dollars 70–95
2024	Mahmoud et al.^130^	1.00–1.40	↑ 10–15%	3.1–3.3	45/65 (at BHCT 212 °F/BHST 284 °F)	<0.5%; excellent	212–284 °F/45 MPa	≈dollars 75–100
2024	Sayed and Hussein^134^	1.05–1.55	↑ 10–16%	3.0–3.4	48/68 (at BHCT 221 °F/BHST 293 °F)	<0.4%; very stable	221–293 °F/45 MPa	≈dollars 80–105
2025	Alharbi et al.^135^	1.00–1.45	↑ 10–18%	3.2–3.5	50/70 (at BHCT 230 °F/BHST 302 °F)	<0.4%; advanced stability (polymeric surf.)	230–302 °F/45 MPa	≈dollars 85–110

**Table 12:** Summary of All Cement Lightweight Additives

additive	type	average cost (USD/ton)	relative to PC (%)	target density (g/cm^3^)	effect on compressive strength	key advantages	economic/technical limitation	row citation
Portland cement (PC)	reference	100	100%	3.15	-	benchmark material	-	-
Zeolite	natural/nano	60–80	60–80%	1.80–2.00	±10%	reduces permeability and ion penetration	slight strength loss at high dosage	Wang et al.^54^
metakaolin (MK)	reactive pozzolan	90–110	90–110%	1.85–2.10	↑15–25%	improved strength, bond, and durability	costly; reduces workability	Siddique and Klaus^70^
vermiculite	lightweight mineral	40–60	40–60%	1.30–1.60	↓10–20%	lightweight, thermal insulation	brittle; lower compressive strength	Koksal et al.^81^
gilsonite	natural bituminous additive	70–90	70–90%	1.50–1.70	↓5–15%	gas-tightness and fluid loss control	limited temperature tolerance (<356 °FS)	Rincon et al.^85^
expanded perlite	siliceous lightweight aggregate	50–70	50–70%	1.20–1.50	↓10–25%	reduced density; good insulation	high water absorption	Celik et al.^90^
ground granulated blast-furnace slag (GGBS)	industrial pozzolan	80–90	80–90%	1.85–2.10	↑10–15%	durability and sulfate resistance	slow setting at low temperature	Siddique^98^
microsphere (HGM)	hollow glass/ceramic particle	40–60	40–60%	1.20–1.40	↓10–20%	uniform lightweight density	fragile; purity-sensitive cost	Wang et al.^107^
cenosphere	fly ash byproduct	30–50	30–50%	1.25–1.35	↓10–15%	low density and high thermal stability	limited supply, variable quality	Danish^111^
silica fume (SF)	nanopozzolan	120–130	120–130%	1.85–2.10	↑20–30%	high strength and bonding	expensive; reduces workability	Mahmoud^123^
foam cement	composite lightweight system	120–150	120–150%	1.00–1.60	design-dependent	ultralight system; pressure control	requires foaming and pumping equipment	Falliano et al.^129^

## 3 Insights Gained

Effectively managing deep wells and weak geological formations poses significant challenges during the well-completion process. One crucial factor in overcoming these challenges is the control of cement density, which is vital for ensuring mechanical stability and providing adequate isolation between the different layers of the formation. Proper cementing techniques are essential for the overall success and safety of the well. Studies indicate that various additives, such as zeolite, metakaolin, vermiculite, gilsonite, perlite (GGBS), microspheres, cenospheres, silica fume, and foam systems, can enhance cement slurries. These additives can achieve densities ranging from 1000 to 2200 kg/m^3^ and compressive strengths of up to 72 MPa. Specifically, the addition of zeolite at 5–25% BWOC and metakaolin at 10–20% improves strength and durability while reducing clinker consumption by over 30%. Moreover, vermiculite and gilsonite contribute to improved thermal resistance and impermeability in the HPHT wells. Vermiculite maintains its mechanical stability up to 900 °C. Perlite enhances the fluidity and adhesion of the slurry by reducing the viscosity by approximately 30% and increasing the yield point by more than three times. Using up to 30% by weight of GGBS strikes an optimal balance between reducing density and retaining strength while also decreasing permeability by about 50%. Both microspheres and cenospheres are effective at densities of 1.2–1.6 g/cm^3^; however, a strength reduction of approximately 10–15% has been reported when their proportion exceeds 30%. Silica fume, at 5–15% BWOC, enhances the long-term stability and sulfate resistance, particularly in CO_2_-rich wells or marine environments. A comprehensive comparison of data shows that the synergistic combination of silica fume with GGBS or metakaolin with zeolite yields the most sustainable lightweight structure. Overall, utilizing these additives can reduce cement consumption and CO_2_ emissions by 25–40%, aligning with the sustainability requirements and practices outlined in APIRP10B-2 and APITR10B-4. Ultimately, optimizing additive compositions under HPHT conditions paves the way for developing safe, environmentally friendly, and cost-effective cementitious materials.
